# SLC27, solute carrier 27 family, a long-chain fatty acid membrane transporters, in human cancers

**DOI:** 10.3389/fcell.2026.1839021

**Published:** 2026-06-22

**Authors:** Zhonghang Xu, Jianshi Du, Wen G. Jiang, Tracey A. Martin

**Affiliations:** 1 Department of Breast Surgery, Jilin University China-Japan Union Hospital, Changchun, China; 2 CCMRC, Cardiff University School of Medicine, Cardiff, United Kingdom

**Keywords:** cancer, FATP, fatty acid activation, fatty acid transport proteins, fatty acid uptake, SLC27

## Abstract

The Solute Carrier family 27 (SLC27), also known as the fatty acid transport proteins (FATPs) is a group of transmembrane integral proteins that facilitate the entry of long-chain and very long-chain fatty acids into cells. The human SLC27 family consists of six highly homologous members (SLC27A1 to A6), which are expressed in various tissues reliant on fatty acids for energy metabolism and biosynthesis, participating in multiple physiological processes. Recent years have seen extensive research on the role of SLC27 in lipid metabolism and more recently in the exploration of the family in the initiation and progression of cancer. Aberrant expression of SLC27s in cancers have been reported, suggesting their potential involvement in key processes such as tumour growth, invasion, metastasis, and immune evasion by promoting fatty acid uptake, remodeling cellular metabolic networks, activating lipid-related signaling pathways, and even modulating the tumour immune microenvironment. Given their significant role in tumor metabolic reprogramming, SLC27s are increasingly regarded as potential therapeutic targets for cancer, attracting widespread attention. The present article summarises the basic characteristics and regulatory mechanisms of the SLC27 family, as well as their cellular functions and roles in cancer, exploring the potential of these family members in tumour metabolism and therapy, thereby providing new directions for future clinical research.

## 1 Background

Long-chain fatty acids (LCFAs), defined as fatty acids with carbon chains exceeding 12 carbon atoms, serve as one of the primary sources of cellular energy supply and are extensively involved in key physiological processes such as cell membrane synthesis, signal transduction, energy metabolism, and the regulation of gene expression. In addition to providing energy for cells, LCFAs also play important roles in maintaining the integrity of cell membrane structures, lipid storage, and lipid signal transduction. Due to their high metabolic demands, tumour cells often rely on large-scale fatty acid uptake to support their rapid proliferation, survival, and invasive behaviors. Therefore, the regulation of fatty acid metabolism is of significant importance in maintaining the malignant phenotype of tumour cells (Zaidi et al., 2013). On the other hand, selective long chain polyunsaturated fatty acids (PUFAs), namely, eicosapentaenoic acid (EPA) and docosahexaenoic acid linolenic acids (DHA) of the ω-3 and gamma linolenic acid (GLA) and dihomogamma linolenic acids (DGLA) of ω-6 are known to be toxic to cancer cells.

The transmembrane transport and intracellular metabolism of LCFAs depend on a series of specialized transport proteins, among which the solute carrier family 27 (SLC27) plays a vital role in the uptake of long-chain fatty acids. This family includes six members (SLC27A1–SLC27A6), each exhibiting specific expression patterns in different tissues and cell types. Their transmembrane structures and acyl-CoA synthetase functions collaboratively participate in the transport and activation of fatty acids (Anderson and Stahl, 2013). By regulating the transmembrane uptake and acylation of fatty acids, SLC27 family proteins not only maintain cellular lipid homeostasis but also profoundly influence the cellular energy metabolism network.

Recent studies have continually revealed the important biological functions of SLC27 family proteins in cancer metabolic reprogramming. Compared with normal cells, tumour cells exhibit significantly altered lipid metabolic characteristics, particularly manifested as enhanced capabilities for fatty acid uptake and utilization to meet the biosynthetic and energy demands of their rapid growth. Members of the SLC27 family show abnormally high expression in various malignant tumors, strongly associated with tumour initiation, progression, and prognosis. They not only act as key mediators of fatty acid transport but also participate in regulating tumour cell growth, invasive capacity, and adaptive responses to metabolic stress by modulating fatty acid metabolic flux. The current article will comprehensively explore the functional roles of various members of the SLC27 family in different types of human cancers and analyses their mechanisms of action in fatty acid transport and metabolism, and their potential involvement in driving cancer initiation, development, and metastasis. Additionally, based on the latest research advances, the feasibility and prospects of targeting SLC27 proteins for metabolic intervention therapy will be evaluated.

### 1.1 Discovery and genetic characteristics of the SLC27 family

The SLC27 family is a class of important transmembrane transport proteins primarily responsible for the transmembrane transport of LCFAs and their derivatives, playing a key role in lipid metabolism. Research on this family began in the late 1990s, with the initial identification of the first member, FAT/CD36, through molecular cloning techniques. This membrane protein mediates the uptake of long-chain fatty acids (Anderson and Stahl, 2013). With advances in genomics and molecular biology research, the composition of the SLC27 family has been gradually clarified, and it is now confirmed to include six highly homologous members, designated SLC27A1 to SLC27A6 (Stahl, 2004) (Table 1). These proteins are all integral membrane proteins, typically containing multiple transmembrane domains that form stable transport channels on the plasma membrane, facilitating the entry of fatty acids from the extracellular space into the cell.

**TABLE 1:** The gene location, subcellular location and functional summary of the SLC27 family.

SLC27 family member	Location of coding gene	Alternative names	Subcellular locations	Tissue distribution
SLC27A1	1p31.1	FATP, FATP1	Cytoplasmic membrane, mitochondria, cytosol	Ubiquitous; highly expressed in adipose tissue, skeletal muscle, heart muscle, and central nervous system
SLC27A2	9q22.33	FATP2, ACSVL1 (Acyl-CoA Synthetase Very Long Chain Family Member 1), VLCS (Very Long-Chain Acyl-CoA Synthetase)	Cytoplasmic membrane, microsomes and peroxisomes	Highly expressed in liver, kidney, and adipose tissue; moderate in small intestine and adrenal gland
SLC27A3	3q13.33	FATP3, VLSC3, ACSVL3	Cytoplasmic membrane, mitochondria, endoplasmic reticulum	Ubiquitous; relatively high in blood vessels, adipose tissue, lung, stomach, and brain
SLC27A4	11q13.5	FATP4, ACSVL4	Cytoplasmic membrane, intracellular vesicles (endoplasmic reticulum)	Ubiquitous; high in digestive organs (esophagus, skin, small intestine, liver)
SLC27A5	6q15	FATP5, ACSVL6, FACVL3 (Fatty acid coenzyme A ligase, very long chain 3), BAL/BACS (bile acid-CoA ligase/bile acid-CoA synthetase)	Cytoplasmic membrane, possible intracellular location	Highly liver-specific (dramatically higher in liver than all other tissues)
SLC27A6	6q22.1	FATP6, FACVL2, ACSVL2	Cytoplasmic membrane, nucleus (possible nuclear bodies)	Highest in heart muscle; also high in adrenal gland, ovary, and retina

**FIGURE 1:**
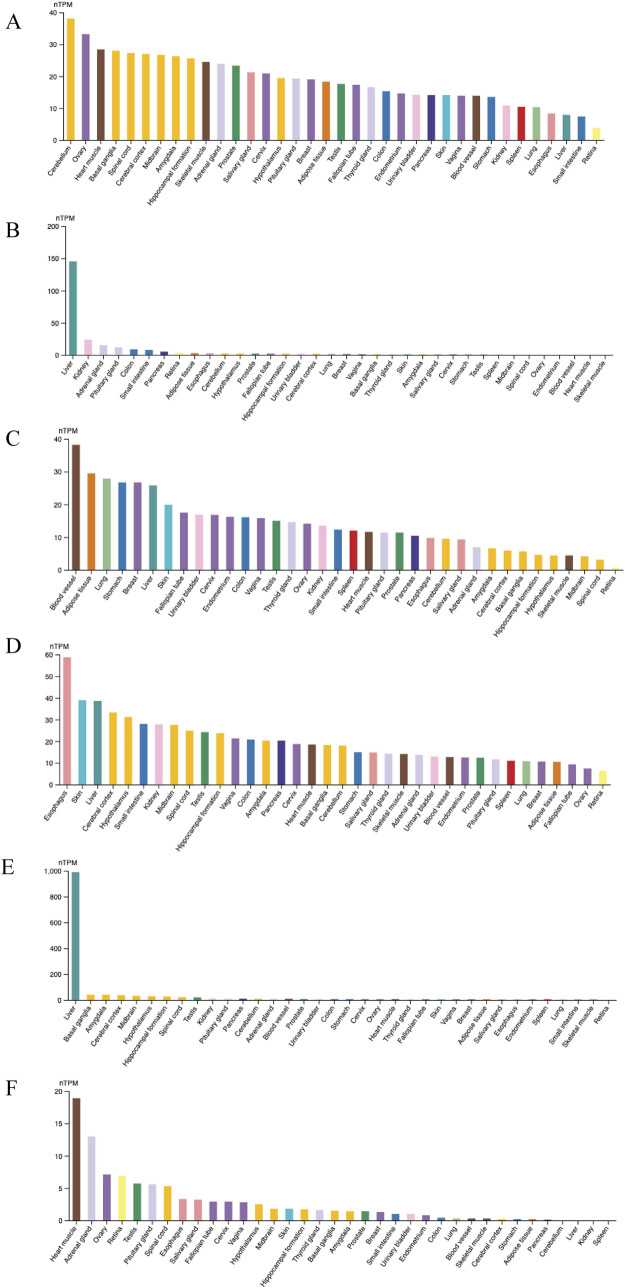
Tissue-specific mRNA expression profile of the SLC27 (FATP) gene family across human tissues. Data are shown as normalized Transcripts Per Million (nTPM) obtained from the Human Protein Atlas (proteinatlas.org) RNA-seq dataset (accessed November 2025). Each panel represents one member of the SLC27 family. Tissues are ordered from highest to lowest expression level for each gene. **(A)** SLC27A1 – Highest expression in cerebellum, ovary, heart muscle, and several brain regions (basal ganglia, spinal cord, cerebral cortex), followed by moderate expression across multiple tissues. **(B)** SLC27A2 – Predominantly expressed in liver (highest), with moderate expression in kidney and lower levels in adrenal gland, pituitary gland, and small intestine. **(C)** SLC27A3 – Broadly expressed with highest levels in blood vessels, adipose tissue, lung, stomach, and brain tissues (cerebellum, cerebral cortex). **(D)** SLC27A4 – Highest expression in esophagus and skin, with prominent levels also in liver, cerebral cortex, and hypothalamus. **(E)** SLC27A5 – Extremely liver-specific, showing dramatically higher expression in liver compared to all other tissues examined (note the different y-axis scale). **(F)** SLC27A6 – Highest expression in heart muscle, with notable levels in adrenal gland, ovary, and retina.

In terms of physiological function, SLC27 family proteins are extensively involved in metabolic processes such as fatty acid uptake, esterification, oxidation, and lipid synthesis, playing a significant role in maintaining intracellular lipid homeostasis and energy balance (Table 1). For example, SLC27A1 and SLC27A2 are abundantly expressed in high-metabolism tissues such as adipose tissue, liver, and skeletal muscle (Figure 1). They promote fatty acid entry into cells and collaborate with acyl-CoA synthetase to complete the acylation activation process of fatty acids, thereby participating in energy metabolism and membrane lipid construction. Additionally, SLC27 proteins also play regulatory roles in fatty acid signal transduction and storage processes, indirectly influencing cellular functional states. For instance, fatty acids, as signaling molecules, can participate in inflammatory responses, insulin sensitivity regulation, and the proliferative behavior of tumour cells. The expression levels of SLC27 family members are regulated by various physiological stimuli, including hormones (such as insulin), inflammatory factors (such as endotoxins, tumor necrosis factor-α(TNF-α), and IL-1), and peroxisome proliferator-activated receptors (PPARs).

From a genetic perspective, the six members of the SLC27 family are located on different chromosomal regions in humans, and the distribution of their genes in the genome exhibits significant heterogeneity (Table 1). This locational difference reflects the functional and regulatory divergence among family members, suggesting that they may have specific functions in different tissues and under various physiological or pathological conditions. Furthermore, increasing evidence indicates that genetic mutations and expression disorders of SLC27 family members are closely associated with the development of various malignant tumors. For example, SLC27A2 is highly expressed in breast cancer and may promote fatty acid uptake and utilization by tumor cells, thereby driving tumour progression. Conversely, SLC27A1 may act as a metabolic suppressor in colorectal cancer, and its downregulation may affect fatty acid metabolic homeostasis, thus participating in the tumorigenesis process. Therefore, the genetic variations and expression states of the SLC27 family may not only serve as potential cancer biomarkers but also provide new insights for metabolic-targeted therapies.

### 1.2 Structure Protein, subcellular localization, and biological functions

The SLC27 family, also referred to as fatty acid transport proteins (FATPs), comprises a highly conserved class of integral transmembrane proteins primarily responsible for the cellular uptake and transmembrane transport of LCFAs and very-long-chain fatty acids (VLCFAs). All SLC27 family members share a conserved core architecture consisting of 12 transmembrane α-helical domains that form a stable membrane-spanning channel, together with a conserved adenosine monophosphate (AMP)-binding motif (Anderson and Stahl, 2013; Stahl, 2004). This dual structural configuration enables SLC27 proteins to function not only as fatty acid transporters but also as acyl-CoA synthetases (ACSs), tightly coupling transmembrane fatty acid import with intracellular activation into acyl-CoA derivatives. The resulting acyl-CoA substrates feed directly into β-oxidation, phospholipid biosynthesis, and lipid-mediated signaling pathways. Importantly, unlike fatty acid scavenger receptors such as CD36, which primarily facilitate passive lipid association at the membrane surface, SLC27 proteins actively regulate the intracellular metabolic fate of imported fatty acids through coordinated coupling with downstream enzymatic machinery (Schaffer and Lodish, 1994; Currie et al., 2013).

#### 1.2.1 Subcellular localization

Members of the SLC27 family exhibit considerable diversity in subcellular localization and are distributed across the plasma membrane, endoplasmic reticulum (ER), peroxisomes, and mitochondria-associated membrane structures (Figure 1; Table 1). This localization heterogeneity is not incidental but is tightly coupled to the tissue-specific expression patterns and distinct biological roles of each member (S et al., 2009; Jia et al., 2007). The localization of a given SLC27 protein determines both its substrate access (exogenous dietary lipids *versus* intracellularly recycled fatty acids) and its functional coupling to specific downstream metabolic compartments.

Plasma membrane-resident members, principally SLC27A1/FATP1 and SLC27A4/FATP4, serve as primary gatekeepers of exogenous LCFA and VLCFA import and are highly expressed in metabolically active tissues including adipose tissue, skeletal muscle, and intestinal epithelium (Schaffer and Lodish, 1994). SLC27A1 is additionally detected at mitochondrial membranes and exhibits dynamic, insulin-stimulated translocation from intracellular storage compartments to the plasma membrane, a mechanism that markedly amplifies LCFA uptake capacity in response to nutrient signals (Stahl et al., 2002; Wu et al., 2006a). This translocation also facilitates direct coupling with carnitine palmitoyltransferase 1 (CPT1) at the mitochondrial outer membrane, thereby channeling imported LCFAs toward β-oxidation (S et al., 2009). SLC27A2/FATP2 is predominantly localized to microsomal and peroxisomal compartments in hepatocytes and renal proximal tubular epithelial cells, consistent with its primary roles in peroxisomal fatty acid activation and VLCFA β-oxidation (Falcon et al., 2010; Uchiyama et al., 1996). Notably, the human SLC27A2 gene encodes two functionally distinct splice variants: FATP2a, which predominantly functions as a very-long-chain acyl-CoA synthetase (VLACS) and is enriched in peroxisomes, and FATP2b, which is oriented toward plasma membrane-mediated fatty acid transport (Melton et al., 2011). This isoform diversity allows SLC27A2 to operate across multiple subcellular compartments and contribute to both transport and activation functions simultaneously. SLC27A4/FATP4 is enriched in the apical membrane and ER of intestinal epithelial cells, positioning it as a critical mediator of dietary fatty acid absorption (Jia et al., 2007). SLC27A5/FATP5 is specifically expressed on the basolateral membrane of hepatocytes, where it mediates LCFA uptake from the portal circulation and additionally functions as a bile acid-CoA ligase (Doege et al., 2006). SLC27A6/FATP6 is primarily localized to the plasma membrane of cardiomyocytes, frequently co-localizing with CD36 to synergistically promote cardiac LCFA uptake (Gimeno et al., 2003). In contrast, the subcellular localization of SLC27A3/FATP3 remains incompletely characterized; current evidence suggests predominant association with the ER or cytoplasm, with potential species-specific differences (Maekawa et al., 2015).

Notably, under pathological conditions—particularly within tumor cells—several SLC27 family members undergo dynamic subcellular relocalization. Such redistribution, for instance to the mitochondrial outer membrane or ER, may represent an adaptive response to heightened metabolic demands for fatty acid flux, contributing to the lipid metabolic reprogramming that characterizes malignancy.

#### 1.2.2 Fatty acid transport function

**FIGURE 2:**
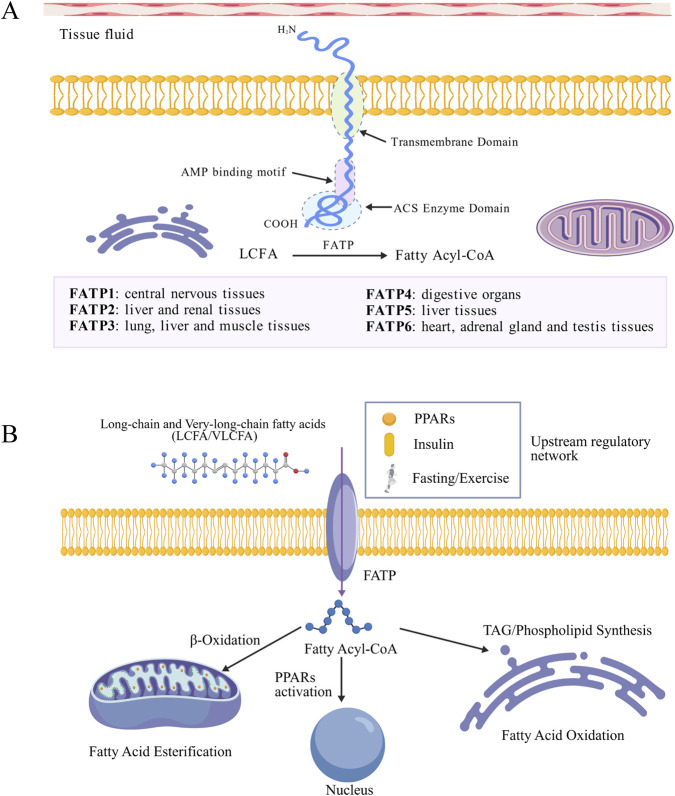
**(A)**. The SLC27 proteins on plasma membrane. **(B)** Transportation of LCFAs by SLC27 proteins.

The principal function of the SLC27 family is to mediate the active transmembrane transport of LCFAs from the extracellular environment into the intracellular space (Figure 2). The conserved 12-transmembrane helical architecture together with the AMP-binding domain constitutes an efficient fatty acid transport system capable of recognizing and internalizing hydrophobic substrates that would otherwise be unable to traverse the lipid bilayer unaided (Anderson and Stahl, 2013; Stahl, 2004; Schaffer and Lodish, 1994).

The transport activity of SLC27 members is subject to dynamic regulation by physiological signals. In adipocytes and skeletal muscle cells, insulin stimulation induces rapid translocation of SLC27A1 from intracellular storage pools to the plasma membrane, significantly enhancing LCFA uptake capacity; knockdown of SLC27A1 under these conditions abolishes insulin-dependent, but not basal, fatty acid transport (Stahl et al., 2002; Wu et al., 2006a). In skeletal muscle, overexpression of SLC27A1 increases LCFA transport rates by approximately 24% and redirects lipid flux toward oxidative pathways by approximately 35%, thereby attenuating triglyceride accumulation (Holloway et al., 2011). At the blood–brain barrier, insulin-stimulated plasma membrane translocation of SLC27A1 is essential for the supply of docosahexaenoic acid (DHA) and other polyunsaturated fatty acids required for normal neural function (Ochiai et al., 2017). SLC27A4 demonstrates a transport efficiency approximately 1.7-fold greater than that of SLC27A1 in skeletal muscle cells, and its activity is further enhanced by short-term insulin stimulation *via* the phosphoinositide 3-kinase (PI3K) signaling pathway—an effect that can be abolished by the PI3K inhibitor wortmannin (Nickerson et al., 2009; Digel et al., 2011).

SLC27A6 plays an important complementary role in fatty acid transport in cardiomyocytes and mammary epithelial cells; its overexpression markedly enhances LCFA uptake, whereas gene silencing significantly suppresses this process (Yen et al., 2019). In bovine mammary epithelial cells, knockdown of SLC27A6 broadly downregulates fatty acid activation and oxidation genes—including ACSL4, CPT1A, and CD36—while concurrently upregulating lipid synthesis and desaturase genes such as PPARG, DGAT1, and FADS2, resulting in significant shifts in intracellular triglyceride content and fatty acid composition, underscoring its governing role in the balance between lipid synthesis and oxidation (Zhang H. et al., 2021).

#### 1.2.3 Acyl-CoA synthetase activity

Beyond their transport function, SLC27 proteins possess intrinsic acyl-CoA synthetase (ACS) activity, enabling the *in situ* activation of imported fatty acids into acyl-CoA derivatives. This coupling mechanism ensures that fatty acids entering the cell are immediately committed to specific metabolic pathways—including β-oxidation, phospholipid biosynthesis, and the generation of lipid signaling molecules—without accumulating as free, potentially cytotoxic species (Ellis et al., 2010).

Among family members, SLC27A1 and SLC27A5 exhibit particularly robust ACS activity and can function synergistically with downstream enzymes such as long-chain acyl-CoA synthetase 1 (ACSL1) to amplify acyl-CoA production (Ellis et al., 2010; Gimeno et al., 2003). SLC27A4 likewise demonstrates significant acyl-CoA synthesis capability; in skin fibroblasts, it serves as the primary activating enzyme for VLCFAs such as C24:0, and its genetic deletion reduces C24:0 activation capacity by 83% and lipid integration efficiency by 54%–64%, with severe consequences for epidermal barrier integrity (Jia et al., 2007). Beyond its role in fatty acid metabolism, SLC27A5 additionally functions as a bile acid-CoA ligase in hepatocytes, catalyzing the conjugation of bile acids with amino acids (glycine and taurine) to facilitate their reabsorption in enterohepatic circulation; silencing of SLC27A5 significantly reduces conjugated bile acid production and increases the proportion of unconjugated primary bile acids (Castro-Perez et al., 2011; Hubbard et al., 2006; Ason et al., 2011).

#### 1.2.4 Interactions with other membrane proteins

SLC27 proteins do not function as isolated transporters but instead operate as components of dynamic, multiprotein complexes at membrane surfaces, through which they coordinately regulate fatty acid uptake, activation, and downstream metabolic routing. These interactions extend the functional repertoire of the SLC27 family well beyond simple lipid translocation.

In cardiomyocytes, SLC27A6 co-localizes with CD36 on the sarcolemma, and this co-complex synergistically enhances LCFA uptake efficiency (Gimeno et al., 2003). SLC27A1 directly interacts with CPT1 at the mitochondrial outer membrane, facilitating the transfer of LCFAs into the mitochondrial matrix for β-oxidation (S et al., 2009). SLC27A2 cooperates with the mitochondrial fusion protein mitofusin 2 (MFN2) and the small GTPase RAB5C to support autophagic flux and mitochondrial homeostasis maintenance under nutrient-deprived conditions (Koundouros and Poulogiannis, 2020; Gordaliza-Alaguero et al., 2025). In melanoma cells, SLC27A2 interacts with fatty acid-binding protein 5 (FABP5) to amplify intracellular lipid storage and promote tumor cell metastatic capacity (Wang et al., 2023); additionally, by modulating acyl-CoA pools, SLC27A2 regulates peroxisome proliferator-activated receptor family signaling in glioma cells, thereby influencing proliferative and apoptotic balance (Grommes et al., 2004). SLC27A5 engages the nuclear factor erythroid 2-related factor 2 (NRF2) signaling pathway to regulate the expression of tyrosine metabolism-associated enzymes, linking lipid metabolic activity to amino acid metabolic networks and influencing hepatocellular cycle progression (Wang et al., 2022). Collectively, these protein–protein interactions demonstrate that the SLC27 family operates as a central node within membrane-associated metabolic complexes, coordinating fatty acid transport with energy homeostasis, mitochondrial function, autophagy, and signal transduction. Disruption of these interaction networks—as observed in various pathological conditions including cancer—may profoundly alter cellular lipid homeostasis and metabolic adaptability.

In summary, the SLC27 family is distinguished by its unique transmembrane architecture, diverse subcellular localization patterns, dual transport-and-activation biochemistry, and extensive capacity for protein–protein interaction. These properties collectively underpin its essential roles in cellular lipid homeostasis and systemic energy balance, and provide a mechanistic foundation for the dysregulation of SLC27 members observed in metabolic disease, cancer, and other pathological conditions.

### 1.3 Physiological functions and non-tumor pathological roles of the SLC27 family

Members of the SLC27 family exhibit distinct tissue-specific expression patterns and collectively maintain systemic lipid homeostasis through the coordinated regulation of LCFA uptake, activation, and intracellular metabolism. Their diverse subcellular localizations underpin functional specialization across tissues—from hepatic fatty acid handling and intestinal lipid absorption to cardiac energy supply and epidermal barrier maintenance. Beyond these physiological roles, dysregulation of SLC27 family members is closely associated with a broad spectrum of non-neoplastic pathological conditions, including obesity, non-alcoholic fatty liver disease (NAFLD), type 2 diabetes mellitus, cardiovascular disease, and chronic inflammatory disorders.

#### 1.3.1 SLC27A1/FATP1

SLC27A1/FATP1, the founding member of the family identified by Schaffer and Lodish in 1994 (Schaffer and Lodish, 1994), is broadly expressed across high-metabolic-demand tissues including adipose tissue, skeletal muscle, cardiac tissue, and blood–brain barrier (BBB) endothelial cells, where it functions as a central regulator of LCFA uptake and utilization (Mitchell et al., 2011; Challa et al., 2015; Wang et al., 2019a). Its expression is subject to multilayered transcriptional and epigenetic regulation. The SLC27A1 gene promoter contains an insulin-response element, and insulin stimulation has been shown to modulate SLC27A1 mRNA levels in white adipose tissue (WAT) (Hui et al., 1998). Conversely, pro-inflammatory cytokines such as TNF-α suppress SLC27A1 expression, attenuating insulin-mediated fatty acid uptake (Stahl et al., 2002). Knockdown of SLC27A1 in 3T3-L1 adipocytes significantly reduces LCFA uptake under both basal and insulin-stimulated conditions, highlighting its indispensable role in adipose metabolic responses (Lobo et al., 2007). Beyond hormonal signals, epigenetic mechanisms also modulate SLC27A1 expression: neonatal supplementation with resveratrol or nicotinamide riboside in mice induces WAT browning while altering SLC27A1 promoter methylation status (Serrano et al., 2020), and this methylation pattern is further linked to systemic homocysteine levels (Mandaviya et al., 2017). In brown adipose tissue (BAT), SLC27A1 expression is upregulated by cold exposure and β3-adrenergic receptor (β3-AR) signaling, accompanied by increased expression of mitochondrial uncoupling protein 1 (UCP1) and enhanced thermogenic capacity (Wu et al., 2006b). Recent evidence further demonstrates that SLC27A1 interacts with the lipid droplet-associated protein perilipin 5 (PLIN5) at membrane contact sites in BAT, facilitating the transfer of fatty acids from lipid droplets to mitochondria for β-oxidation—a mechanism critical for non-shivering thermogenesis (Miner et al., 2023).

Under pathological conditions, the significance of SLC27A1 becomes particularly evident. In skeletal muscle, genetic deletion of Slc27a1 protects mice from high-fat diet-induced lipid accumulation and insulin resistance (Kim et al., 2004), while its overexpression in obese patients promotes adipocyte hypertrophy and ectopic lipid deposition in non-adipose tissues, disrupting insulin signaling (Chiu et al., 2023; Kazantzis and Stahl, 2011). SLC27A1 is also implicated as a key mediator of exercise-induced WAT browning and synergizes with the PPAR signaling pathway to enhance fatty acid oxidation, with its expression increasing after weight loss in parallel with improved insulin sensitivity (Challa et al., 2015; Wang et al., 2019a). Cardiac-specific overexpression of SLC27A1 results in an approximately fourfold increase in myocardial free fatty acid uptake, leading to lipid accumulation, diastolic dysfunction, and a phenotype resembling lipotoxic cardiomyopathy (Chiu et al., 2005). Within the central nervous system, SLC27A1 is the primary LCFA transporter in BBB endothelial cells, and its knockdown significantly impairs DHA transport across the barrier (Mitchell et al., 2011). In Alzheimer’s disease models, amyloid-β (Aβ) downregulates SLC27A1 expression, reducing DHA efflux across the BBB (Ochiai et al., 2019); iron overload similarly modulates SLC27A1 activity to impair DHA transport capacity (Supti et al., 2024), collectively highlighting therapeutic opportunities in both metabolic and neurodegenerative contexts.

#### 1.3.2 SLC27A2/FATP2

SLC27A2/FATP2 is predominantly expressed in the liver, with lower levels detected in the kidney and placental trophoblast cells, and is nearly undetectable in other tissues (Falcon et al., 2010). Unlike most SLC27 family members, SLC27A2 possesses dual functionality: it operates as both a plasma membrane fatty acid transporter and a peroxisomal VLACS (Falcon et al., 2010; Uchiyama et al., 1996). This functional duality is structurally supported by the existence of two splice variants of the human SLC27A2 gene—FATP2a, which predominantly functions as a VLACS and is enriched in peroxisomes, and FATP2b, which is primarily oriented toward plasma membrane-mediated fatty acid transport—with multiple subcellular localizations across the peroxisome, endoplasmic reticulum, and plasma membrane further supporting this diversity (Melton et al., 2011). Under physiological conditions, SLC27A2 plays essential roles in hepatic lipid homeostasis, intestinal stem cell maintenance, and reproductive biology. In hepatocytes, SLC27A2 dynamically interacts with the tetraspanin TM4SF5 to regulate acute fatty acid uptake and maintain cellular energy homeostasis (Park et al., 2021). In the intestine, SLC27A2 supports the self-renewal of intestinal epithelial stem cells through activation of fatty acid oxidation-related genes (including Acsl5, Acsf2, Fabp2, and Hadh); double knockout of Hnf4a and Hnf4g leads to marked SLC27A2 downregulation, reduced fatty acid oxidation (FAO) activity, and impaired tricarboxylic acid cycle intermediate production (Chen et al., 2020). SLC27A2 is also highly expressed in epididymal clear cells, where it cooperates with epididymosomes to deliver lipid-associated proteins to the sperm surface, supporting lipid-dependent sperm maturation with significant regional specificity (Hermoso et al., 2020).

In non-neoplastic diseases, SLC27A2 is upregulated in NAFLD models, and its knockdown markedly reverses hepatic steatosis and improves insulin sensitivity, establishing it as a promising therapeutic target (Falcon et al., 2010). In gallbladder epithelial cells, SLC27A2 mediates fatty acid uptake leading to ectopic triacylglycerol accumulation and gallbladder hypomotility; adeno-associated virus-mediated knockdown of SLC27A2 significantly reduces triglyceride content and restores contractile function without affecting biliary cholesterol saturation (Tharp et al., 2016). In the kidney, SLC27A2 localizes to the apical membrane of proximal tubular epithelial cells, mediating albumin-bound non-esterified fatty acid reabsorption; its genetic deletion reduces lipid uptake and attenuates lipotoxicity-induced tubular apoptosis (Khan et al., 2018; Khan et al., 2020a). SLC27A2 expression is also dysregulated in heart failure with preserved ejection fraction (HFpEF), where it contributes to myocardial metabolic reprogramming; low-frequency papillary muscle electrical stimulation can partially reverse this aberrant expression by improving insulin signaling and oxidative phosphorylation (Chakraborty et al., 2024). At the systemic level, epigenetic silencing of SLC27A2 in the subcutaneous adipose tissue of low-birthweight males is associated with insulin resistance and elevated type 2 diabetes risk (Gillberg et al., 2016), while in primary hepatocytes, SLC27A2 synergizes with FAT/CD36 to mediate palmitate- and oleate-induced insulin resistance through diacylglycerol and ceramide accumulation that inhibits inhibits AKT/glycogen synthase kinase 3 (GSK3) signaling (Chabowski et al., 2013).

#### 1.3.3 SLC27A3/FATP3

SLC27A3/FATP3, encoded by the SLC27A3 gene, is one of the less extensively characterized members of the SLC27 family. It is broadly expressed across the liver, lung, adipose tissue, mammary gland, placenta, and neural stem cells (Maekawa et al., 2015). Physiologically, SLC27A3 mediates LCFA transmembrane transport and acyl-CoA activation, participates in lipid droplet metabolism, and regulates autophagic processes. Its transcription is promoted by signal transducer and activator of transcription 2 (STAT2), which binds directly to the SLC27A3 promoter—a regulatory relationship confirmed by dual-luciferase reporter assays (Viraragavan et al., 2021; Lu D. et al., 2024). Under conditions of dietary fat excess, SLC27A3 expression in visceral adipose tissue is markedly upregulated (approximately 9.6-fold) in parallel with reductions in promoter DNA methylation, reflecting its epigenetically mediated induction under metabolic stress (Viraragavan et al., 2021).

In non-neoplastic pathological conditions, SLC27A3 contributes to disease progression across several organ systems. In the lung, SLC27A3 expression is significantly elevated in murine models of chronic obstructive pulmonary disease (COPD). and is closely associated with impairment of lung function and exaggerated inflammatory responses. Machine learning-based analyses have identified SLC27A3 together with STAU1 as candidate COPD biomarkers, with expression patterns linked to immune cell infiltration profiles (Zhang et al., 2022). Mechanistic studies confirm that SLC27A3 promotes Th17/regulatory T-cell (Treg) imbalance through activation of the JAK2/STAT3 signaling pathway, and its knockdown significantly alleviates pulmonary inflammation (Li et al., 2025). In the kidney, SLC27A3 aggravates proximal tubular lipotoxic injury in diabetic nephropathy (DKD) by promoting excessive LCFA uptake; aberrant CpG methylation patterns at the SLC27A3 locus in renal tissues of DKD patients are further correlated with disease progression, implicating epigenetic dysregulation in renal lipid metabolic imbalance (Khan et al., 2020b; Akhouri et al., 2023). In placental tissue, SLC27A3 expression correlates positively with maternal pre-pregnancy body mass index, suggesting a role in modulating fetal lipid supply under conditions of maternal metabolic overload (Hirschmugl et al., 2017). Genetic association studies in the Japanese population have further identified multiple rare and low-frequency nonsynonymous variants in SLC27A3 significantly associated with increased stature (P_SKAT-O < 2.5 × 10^−6^, explaining 1.7% of phenotypic variance), implying potential indirect effects on skeletal development through modulation of lipid metabolism (Akiyama et al., 2019).

#### 1.3.4 SLC27A4/FATP4

SLC27A4/FATP4 is primarily localized to the apical membrane of intestinal epithelial cells and the skin, while also being distributed in skeletal muscle, uterine tissue, and placental tissue (Korbecki et al., 2023). Its expression levels are regulated by nutritional and hormonal status: in the pregnant bovine uterus, SLC27A4 expression is higher than during estrus and is further induced by polyunsaturated fatty acids such as arachidonic acid (Blitek and Szymanska, 2024), while high-fat diet feeding significantly upregulates SLC27A4 in the murine small intestine (Torelli Hijo et al., 2019). Physiologically, SLC27A4 serves dual functions as a VLCFA transporter and an acyl-CoA synthetase, mediating both dietary fatty acid absorption and the activation of VLCFAs for incorporation into structural lipids.

In the skin, SLC27A4 is indispensable for epidermal barrier integrity. It regulates the incorporation of VLCFAs (such as C26:0 and C26:0-OH) into sphingolipids and monoacylglycerols that constitute the stratum corneum lipid matrix (Jia et al., 2007; Schmuth et al., 2005a). Genetic deletion of Slc27a4 in mice results in severe epidermal hyperkeratosis, skin stiffening, and neonatal lethality—a phenotype mechanistically linked to ectopic activation of the EGFR-STAT3 signaling axis, which can be partially rescued by EGFR inhibitor treatment (Lin et al., 2010). In humans, loss-of-function mutations in SLC27A4 (including nonsense mutations such as p.C168X and missense mutations such as p.V477D and p.R504H) cause ichthyosis prematurity syndrome, validating its critical role in human epidermal lipid metabolism (Klar et al., 2009). Transgenic rescue experiments further confirm that restoration of SLC27A4 with intact acyl-CoA synthetase activity is sufficient to correct the skin phenotype (Moulson et al., 2007). In the intestine, SLC27A4 is the primary mediator of brush-border dietary fatty acid uptake. Keratinocyte-specific overexpression of SLC27A4 in knockout mice rescues the skin phenotype without affecting intestinal cholesterol absorption, demonstrating tissue-specific functional independence (Shim et al., 2009). Intestinal epithelial-specific Slc27a4 deletion reduces ileal VLCFA and sphingolipid/phospholipid content while increasing neutral lipids, indicating a key role in regulating the balance between polar and neutral lipid metabolism in the gut (Seessle et al., 2024). SLC27A4 in intestinal L cells additionally mediates oleic acid-induced glucagon-like peptide-1 (GLP-1) secretion, and its knockout significantly reduces postprandial GLP-1 levels (Poreba et al., 2012). In retinal pigment epithelial (RPE) cells, SLC27A4 acts as a negative regulator of RPE65 isomerase by competing for substrate, thereby protecting photoreceptor cells from light-induced degeneration (Li S. et al., 2013). In skeletal muscle, SLC27A4 promotes uptake of both saturated and unsaturated LCFAs with a transport efficiency approximately 1.7-fold greater than SLC27A1, and its activity is further enhanced by insulin *via* PI3K signaling (Nickerson et al., 2009; Digel et al., 2011; Benninghoff et al., 2020).

#### 1.3.5 SLC27A5/FATP5

SLC27A5/FATP5 is the most tissue-restricted member of the SLC27 family, with expression confined almost exclusively to the liver (Figure 1), where it localizes to the basolateral plasma membrane of hepatocytes (Doege et al., 2006). This highly specific localization is consistent with its dual physiological roles as the primary mediator of portal LCFA uptake from the bloodstream and as a bile acid-CoA ligase that catalyzes the conjugation of primary bile acids with amino acids (glycine and taurine) to facilitate their reabsorption and enterohepatic circulation (Castro-Perez et al., 2011; Hubbard et al., 2006; Ason et al., 2011). *In vitro* overexpression of SLC27A5 markedly enhances ^14^C-oleic acid uptake capacity, while hepatocytes from Slc27a5 knockout mice exhibit significantly reduced LCFA transport, confirming its non-redundant role in hepatic fatty acid handling (Doege et al., 2006). Following SLC27A5 deletion, intrahepatic triglyceride and free fatty acid levels decrease, accompanied by compensatory upregulation of *de novo* lipogenesis enzymes and redistribution of lipids toward adipose tissue, reflecting the breadth of its impact on systemic lipid homeostasis (Doege et al., 2006; Hubbard et al., 2006). In Slc27a5 knockout mice under high-fat diet feeding, reduced weight gain despite normal fat absorption suggests that SLC27A5 exerts additional regulatory effects on energy expenditure and food intake beyond its transport function (Hubbard et al., 2006). Regarding its bile acid-specific role, SLC27A5 knockout mice produce bile predominantly composed of unconjugated primary bile acids with a marked reduction in secondary bile acids, establishing SLC27A5 as the dominant enzyme for hepatic bile acid re-conjugation rather than *de novo* synthesis (Hubbard et al., 2006; Ason et al., 2011; Falany et al., 2002).

In non-neoplastic diseases, SLC27A5 expression is significantly elevated in liver tissues of early NAFLD patients. A functional variant in the SLC27A5 promoter (A allele) is associated with hepatic steatosis, elevated postprandial insulin and triglyceride levels, and increased ALT activity, suggesting that enhanced SLC27A5-mediated LCFA uptake contributes to hepatic lipid accumulation and progression to insulin resistance (Mitsuyoshi et al., 2009). Genetic ablation of SLC27A5 ameliorates high-fat diet-induced weight gain and markedly alters bile acid composition and enterohepatic circulation (Hubbard et al., 2006; Ason et al., 2011), while its potential to indirectly modulate transmembrane cholesterol transport has implications for cholesterol dysregulation-associated conditions such as atherosclerosis.

#### 1.3.6 SLC27A6/FATP6

SLC27A6/FATP6 is primarily localized to the plasma membrane of cardiomyocytes and adjacent cardiac microvascular endothelial cells, where it cooperates with CD36 to facilitate LCFA uptake and sustain the exceptionally high energetic demands of the working myocardium (Gimeno et al., 2003). Under physiological conditions, SLC27A6 is essential for myocardial lipid metabolic maturation and contractile function, as demonstrated by knockdown studies in human induced pluripotent stem cell-derived cardiomyocytes, which result in immature transcriptional profiles, impaired calcium handling, and reduced contractility (Zhu W. et al., 2023). Beyond the heart, SLC27A6 is additionally expressed in mammary epithelial cells, participating in local fatty acid metabolism regulation (Zhang H. et al., 2021), as well as in hair follicle epithelium (Schmuth et al., 2005b) and placental trophoblast cells (Mishima et al., 2011), suggesting potential roles in epidermal development and maternal–fetal lipid nutrient transport.

In non-neoplastic pathological conditions, SLC27A6 expression is markedly downregulated in rat myocardial infarction models, accompanied by impaired fatty acid β-oxidation capacity and reduced lipid uptake—changes that may exacerbate post-infarction myocardial metabolic dysfunction (Heather et al., 2006a; Heather et al., 2006b). A T>A single-nucleotide polymorphism in the 5′untranslated region (rs2526246) of SLC27A6 is associated with lower fasting and postprandial triglyceride concentrations, reduced blood pressure, and decreased risk of left ventricular hypertrophy, suggesting a cardioprotective metabolic phenotype conferred by this variant (Li J. et al., 2013; Auinger et al., 2012). The AA homozygous genotype specifically correlates with lower insulin concentrations and reduced left ventricular myocardial mass (P = 0.04), indicating that SLC27A6 genetic variation may influence cardiovascular phenotypes and metabolic syndrome risk by modulating myocardial fatty acid metabolism (Auinger et al., 2012).

In summary, members of the SLC27 family collectively maintain systemic lipid homeostasis through tissue-specific expression and functionally complementary roles spanning hepatic lipid handling (SLC27A2, SLC27A5), intestinal absorption and epidermal integrity (SLC27A4), skeletal muscle and adipose energy metabolism (SLC27A1), cardiac fuel supply (SLC27A6), and broader metabolic adaptation across multiple tissues (SLC27A3). Under non-neoplastic pathological conditions, aberrant expression or loss of function of these proteins broadly underpins the development and progression of metabolic syndrome, NAFLD, diabetic complications, cardiovascular disease, and chronic inflammatory disorders.

### 1.4 Roles of the SLC27 family in cancer

**FIGURE 3:**
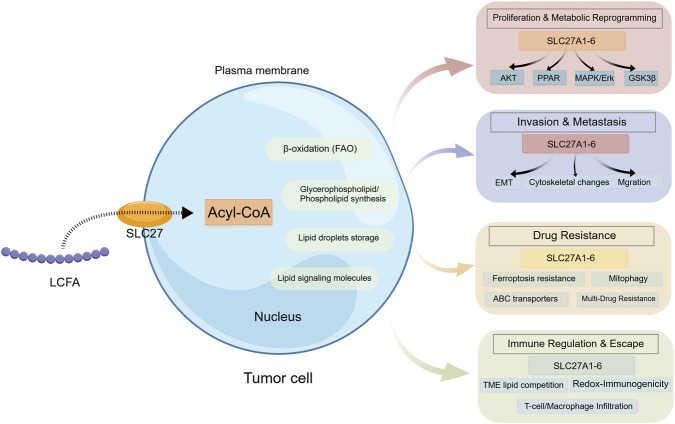
Overall mechanisms of action of the SLC27 family in tumor cells.

As established above, dysregulation of SLC27 family members is a recurrent feature of non-neoplastic metabolic diseases. In the oncological context, these disruptions are markedly amplified and mechanistically distinct: tumor cells exploit SLC27-mediated fatty acid uptake not merely to sustain homeostatic lipid balance, but to actively drive metabolic reprogramming, support aggressive growth, enable invasion, evade therapy, and remodel immune surveillance. The overall mechanisms of action of the SLC27 family in tumor cells are illustrated in Figure 3. Cancer cells are characterized by dramatically elevated demands for energy production, membrane biosynthesis, and lipid-derived signaling molecules, all of which depend on sustained LCFA supply (Zaidi et al., 2013; Currie et al., 2013). As key mediators of LCFA import and intracellular activation, members of the SLC27 family are broadly implicated in tumor initiation, progression, metastasis, therapeutic resistance, and immune escape. Increasing evidence indicates that aberrant SLC27 expression is closely associated with tumor metabolic adaptability, malignant phenotypes, and treatment responsiveness. Beyond supporting anabolic lipid metabolism, SLC27 proteins coordinate FAO, lipid storage, oxidative stress responses, and metabolic signaling networks, thereby reshaping the overall biological behavior of cancer cells. The clinical expression profiles of SLC27 members across major cancer types are summarized in Table 2.

**TABLE 2:** SLC27 family in clinical solid cancers.

SLC family member	Cancer type	Study protocol	Findings	References
SLC27A1	Ovarian cancer	Bioinformatics based analysis	Significant reduction in ovarian cancer	Chen et al. (2021)
	Liver hepatocellular cell carcinoma (HCC)	Bioinformatics based analysis and IHC (n = 261)	High protein and transcript levels are linked with favorable RFS but not OS	Li et al. (2023a)
SLC27A2	Ovarian cancer	Bioinformatics based analysis	Linked with poor overall survival	Chen et al. (2021)
	Pancreatic cancer	Gene transcript analysis (n = 24)	Highly expressed in PDAC than normal tissues	Poenaru et al. (2025)
	Breast cancer	Immunohistochemistry (IHC) (n = 80)	30% of the long-term survivors and 20% of the short-term survivors expressed SLC27A2 in the cell cytoplasm. No significant link with disease specific survival	Shubbar et al. (2013)
	Lung cancer	Bioinformatic analysis	Reduced in lung cancers but not link with prognosis	Liu et al. (2018)
	Non-functional pituitary tumors	​	Downregulated in invasive pituitary tumors	Wang et al. (2019b)
	Thyroid cancer	Transcript and protein (IHC) analyses (n = 98)	High levels of protein in thyroid tumors and in tumours with lymph node metastasis and high stage. It is correlated with clinical prognosis	Feng et al. (2022)
	Renal clear cell Carcinoma (RCCC)	Bioinformatics and protein blotting and IHC	Tumors had low levels of protein. High levels are favorable indicator for DFS and OS. Low level is a risk factor for metastasis	Xu et al. (2022)
	Liver HCC	Bioinformatic analysis	Reduced in liver tumour tissues compared with normal liver.	AmeliMojarad et al. (2024)
		Compound based	The protein is able to convert a small compound inhibitor of ACLY and exert anticancer functions.	Gautam et al. (2025)
SLC27A3	Pancreatic cancer	Gene transcript analysis (n = 24)	Highly expressed in PDAC than normal tissues and linked to lymph node metastasis	Poenaru et al. (2025)
	Pleomorphic Sarcomas and Leiomyosarcomas	Quantitative transcript analysis	Significantly high levels in both Pleomorphic Sarcomas (n = 11) and Leiomyosarcomas (n = 16)	Silveira et al. (2013)
	Colorectal cancer	Quantitative transcript analysis (N = 39)	Transcript increased in tumour tissues compared with normal tissues adjacent to tumors	Niculae et al. (2022)
	Renal RCC	Bioinformatics analysis and IHC	Transcript levels are high in tumour tissues and high levels leading to short OS, DSS and PFS	Lu et al. (2024a)
SLC27A4	Ovarian cancer	Bioinformatics based analysis	Linked with poor overall survival	Chen et al. (2021)
	Pancreatic cancer	Gene transcript analysis (n = 24)	Highly expressed in PDAC than normal tissues	Poenaru et al. (2025)
	Lung cancer	Bioinformatics based analysis	High levels Linked with poor OS	(Liu et al., 2018), (Zheng et al., 2023)
	Breast cancer	Bioinformatics based analysis and IHC	Breast cancer had high levels of expression and high levels Linked with poor OS and poor DMFS	Yen et al. (2018)
	Renal clear cell carcinoma	IHC (n = 180)	The membrane staining was seen high in tumour cells, in high grated and high stage tumors. High staining levels are linked with high incidence of recurrence and recurrence free survival.	Kim et al. (2019)
	Bladder cancer	IHC (n = 286)	Over 47% tumors overexpress the protein and high expression seen in high grade and advanced stage and linked to a poor overall survival	Jeong et al. (2021)
​	Liver HCC	Bioinformatics analysis	High transcript levels are linked with shorter OS and RFS	(Li et al., 2023a), (Yeung et al., 2025)
	Colorectal cancer	Bioinformatics analysis	Tumour tissues expressed low levels, but a high level is linked with a poor OS	Yang et al. (2024)
		Quantitative transcript analysis (N = 39)	Transcript decreased in tumour tissues compared with normal tissues adjacent to tumors	Niculae et al. (2022)
SLC27A5	Ovarian cancer	Bioinformatics based analysis	Linked with poor overall survival	Chen et al. (2021)
	Esophageal SCC	Bioinformatics	Low levels indicating poor clinical outcomes	Guo et al. (2016)
	Hepatocellular carcinoma (HCC)	IHC (n = 40) and bioinformatic analysis.	Reduced levels lead to poor prognosis. More reduction in tumors with poor differentiation and early recurrence. Transcript analysis showing raised SLC27A5 is linked with favorable overall survival and disease-free survival.	(Gao et al., 2020), (Zhang et al., 2021b), (Liu et al., 2024)
	Breast Cancer	Transcriptomic analysis	Expression levels linked with poor disease-free survival and distant metastasis.	Xu et al. (2025)
	Esophageal cancer	Bioinformatics analysis	Expression levels are linked with favorable prognosis and patient’s response to oxaliplatin	Cui et al. (2024)
​	Lung cancer	Bioinformatics based analysis	High levels Linked with poor OS	Liu et al. (2018)
SLC27A6	Ovarian cancer	Bioinformatics and IHC (n = 120)	Significant reduction in ovarian cancer tissues and linked to poor overall survival	Chen et al. (2021)
	Breast cancer	Bioinformatics analysis	Low levels of expression in breast tumour tissues	Yen et al. (2019)
	Prostate cancer	Transcriptomic analysis on prostate cancer cells	Levels are associated with drug resistance of prostate cancer cells	Verma et al. (2020)
	Thyroid cancer	IHC (n = 58)	High level expression on papillary thyroid cancer than normal tissues and particularly in those with *BRAF* mutation	Liu et al. (2022)
	Colorectal cancer	Bioinformatics and IHC (n = 54)	Reduction in transcript expression in cancer but not in protein expression	Gao et al. (2025)
	Lung cancer	Bioinformatics based analysis	High levels marginally Linked with poor OS	Liu et al. (2018)

#### 1.4.1 SLC27 family members in tumor metabolic reprogramming and proliferation

One of the defining hallmarks of cancer is metabolic reprogramming, wherein tumor cells rewire energy metabolism and biosynthetic pathways to sustain rapid proliferation under nutrient-fluctuating and often hypoxic conditions (Zaidi et al., 2013). The SLC27 family contributes substantially to this process by facilitating LCFA uptake and intracellular activation, thereby fueling β-oxidation, membrane phospholipid biosynthesis, and lipid-derived second messenger signaling. A key upstream regulator of this process is hypoxia-inducible factor-1α (HIF-1α), which under tumor hypoxia transcriptionally upregulates SLC27A1 and SLC27A2, thereby enhancing LCFA import and enabling sustained energy supply through FAO even in oxygen-limited environments (Zhang et al., 2023). Additionally, epigenetic alterations of SLC27 family members—including promoter hypomethylation and gene body hypermethylation—are closely associated with tumor immune reactivity and chemoresistance (Nath et al., 2021), suggesting that the SLC27 family operates as both a metabolic driver and an epigenetically regulated determinant of tumor phenotype. Collectively, these upstream regulatory mechanisms establish the foundation for the member-specific oncogenic functions described below.

Among the SLC27 family, SLC27A1 has been most extensively implicated in tumor lipid metabolic remodeling. In melanoma, SLC27A1 is significantly overexpressed compared with other SLC27 family members and drives disease progression by mediating lipid transfer from stromal adipocytes to tumor cells, a mechanism that can be pharmacologically disrupted by the small-molecule inhibitor Lipofermata to reduce tumor growth and invasiveness (Zhang et al., 2018). In invasive pituitary adenomas, SLC27A1 expression is markedly elevated relative to non-invasive primary adenomas, as validated by mRNA sequencing and immunohistochemistry; notably, temozolomide treatment suppresses SLC27A1 expression in pituitary adenoma cell lines, suggesting a potential role in regulating invasive behavior (Cheng et al., 2022). In breast cancer, SLC27A1 is transcriptionally regulated by estradiol/estrogen receptor β (ER-β), and pharmacological modulation of ER-β activity using the arylpiperazine inhibitor DS22420314 effectively suppresses SLC27A1-mediated LCFA uptake and reduces tumor cell viability across multiple breast cancer subtypes (Mendes et al., 2019). In pancreatic ductal adenocarcinoma (PDAC), SLC27A1 is significantly upregulated compared with non-tumor tissues (fold change = 5.66, P = 0.033), indicating active involvement in tumor-associated lipid metabolic reprogramming (Poenaru et al., 2025). SLC27A1 further participates in mitochondrial metabolic pathways supporting the aberrant energetic phenotype of high-grade serous ovarian cancer, where its expression is linked to copy number variations (Meng et al., 2023).

SLC27A2 exerts broad oncogenic regulatory functions across hematological and solid tumors. CEACAM6-mediated upregulation of SLC27A2 promotes its interaction with the deubiquitylase USP29, enhancing SLC27A2 protein stability, LCFA uptake capacity, and FAO flux; pharmacological inhibition of SLC27A2 correspondingly attenuates tumorigenic capacity (Mao et al., 2025). In ovarian cancer, sulfosuccinimidyl oleate (SSO)-mediated blockade of SLC27A2 suppresses LCFA uptake and induces dose- and time-dependent inhibition of proliferation, cell cycle arrest, and apoptosis, demonstrating a high degree of lipid supply dependency in this tumor type (Lemberger et al., 2022). In MYCN-amplified high-risk neuroblastoma, MYCN directly transcriptionally activates SLC27A2, promoting LCFA uptake and glycerophospholipid biosynthesis to sustain tumor cell survival; genetic deletion or pharmacological inhibition of SLC27A2 severely impairs tumor cell viability and synergizes with conventional chemotherapy in preclinical models (Tao et al., 2022). In acute lymphoblastic leukemia (ALL) Jurkat cells, SLC27A2 knockdown suppresses proliferation, reduces Akt pathway activity, and decreases B-cell proportion (Lu L. et al., 2024). In diffuse large B-cell lymphoma (DLBCL), elevated SLC27A2 expression positively correlates with CD4^+^ and CD8^+^ T-cell infiltration and macrophage abundance, participating in lipid metabolism, immune regulation, and cell cycle control; conversely, in acute myeloid leukemia (AML), SLC27A2 expression is relatively low and negatively correlates with CD8^+^ T-cell and B-cell infiltration (Wang et al., 2024). In colorectal cancer (CRC), SLC27A2 is markedly overexpressed in tumor tissues, synergistically regulating LCFA uptake and β-oxidation through PPAR signaling while activating non-genomic pathways involving p-Erk/Erk and p-GSK3β/GSK3β, and modulating ABCD3 expression to sustain lipid metabolic reprogramming (Shang et al., 2023).

SLC27A3 contributes to tumor metabolic adaptation principally through its acyl-CoA synthetase activity, which promotes fatty acid activation and supports plasma membrane biosynthesis and second messenger lipid signaling. In glioma, SLC27A3 maintains oncogenic properties through AKT pathway activation (Silveira et al., 2013). In PDAC, SLC27A3 is significantly upregulated (FC = 2.68, P = 0.040) and cooperates with SLC27A2, SLC27A4, and acyl-CoA synthetases ACSL1 and ACSL3 to drive abnormal lipid synthesis and metabolic remodeling (Poenaru et al., 2025). In glioblastoma, SLC27A3 expression remains relatively preserved compared with the decreased expression of SLC27A4 and SLC27A6, and positively correlates with the fatty acid elongase ELOVL6, suggesting its continued functional role in glioblastoma lipid metabolism (Korbecki et al., 2023).

SLC27A4 exhibits context-dependent functions across tumor types. In HCC, SLC27A4 is significantly overexpressed, particularly in fatty acid-rich microenvironments, where it selectively transports monounsaturated fatty acids (MUFAs) and increases their incorporation into phosphatidylcholine and phosphatidylethanolamine, thereby enhancing membrane resistance to lipid peroxidation and ferroptosis; high SLC27A4 expression is associated with shorter overall survival and relapse-free survival (Li Z. et al., 2023). In breast cancer, SLC27A4 is upregulated across multiple molecular subtypes; silencing of SLC27A4 suppresses EMT, reduces the expression of Slug, vimentin, and α-smooth muscle actin, inhibits LCFA uptake, and induces G2/M cell cycle arrest in Hs578T and MDA-MB-231 cells (Yen et al., 2018). In contrast, SLC27A4 expression is significantly reduced in glioblastoma relative to adjacent brain tissue, accompanied by impaired fatty acid uptake (Korbecki et al., 2023), underscoring the context-dependency of its function.

In marked contrast to the predominantly oncogenic roles of SLC27A1–A4, SLC27A5 frequently exhibits tumor-suppressive characteristics, particularly in liver cancer. SLC27A5 expression is generally low in HCC and is subject to transcriptional silencing through promoter DNA methylation. Low SLC27A5 expression is significantly associated with tumor progression, advanced pathological stage, and poor prognosis, serving as an independent risk factor for unfavorable outcomes in HCC patients (HR = 2.0, 95% CI = 1.4–2.9) (Gao et al., 2020; Lee et al., 2022). Mechanistically, SLC27A5 suppresses HCC metastasis through a non-canonical pathway: it interacts with IGF2BP3, preventing IGF2BP3 nuclear translocation, thereby inhibiting PIP4K2A pre-mRNA splicing and reducing PIP4K2A-S isoform levels, ultimately suppressing PI3K signaling (Gao et al., 2020). SLC27A5 also inhibits HCC cell proliferation and migration and upregulates the cuproptosis regulator FDX1, implicating it in copper metabolism-related tumor suppression (Li X. et al., 2023). Hypoxia further represses SLC27A5 expression by inhibiting HNF4A, thereby activating AKT and CDK2/CCNE1 to promote G1-to-S phase transition and tumor cell proliferation; combination therapy with an HNF4A agonist and an AKT inhibitor synergistically suppresses tumor growth (Tao et al., 2025). It is important to note that SLC27A5’s tumor-suppressive profile is not universal: in lung cancer, high SLC27A5 expression has been paradoxically associated with poor clinical outcomes (Liu et al., 2018), indicating that its biological role is shaped by the tumor microenvironment and organ-specific metabolic context.

SLC27A6 displays a tissue-context-dependent role in cancer. Its expression is higher in non-tumor breast tissue than in breast cancer, and knockdown of SLC27A6 in non-tumor breast cells (H184B5F5/M10) significantly inhibits LCFA uptake and proliferation, reducing CDK4, CDK6, and Cyclin D1 expression and delaying cell cycle progression; however, in tumor breast cells with intrinsically low SLC27A6 expression, such knockdown produces no discernible phenotypic effect (Uchiyama et al., 1996). In KrasG12D mutant mice treated with the CCK2R antagonist JNJ-26070109, downregulation of SLC27A6 is accompanied by an 88% reduction in PDAC incidence (P < 0.004), implicating SLC27A6 in the metabolic regulation of pancreatic tumorigenesis (Mohammed et al., 2019).

Taken together, these findings reveal a spectrum of functional roles within the SLC27 family in tumor metabolic reprogramming: SLC27A1, A2, A3, and A4 predominantly function as oncogenic drivers of lipid import and metabolic rewiring, whereas SLC27A5 primarily acts as a tumor suppressor in hepatic cancers, and SLC27A6 displays context-dependent roles that differ between normal and malignant tissue. This functional heterogeneity must be considered when evaluating the SLC27 family as a therapeutic target.

#### 1.4.2 Roles of the SLC27 family in tumor invasion and metastasis

Beyond their contributions to intratumoral metabolic reprogramming, SLC27 family members promote invasion and metastasis through coordinated regulation of lipid-derived signaling, EMT, and cytoskeletal reorganization. While Section 4.1 addressed the roles of SLC27 members in sustaining primary tumor growth, this section focuses specifically on their contributions to the acquisition of invasive and migratory phenotypes—processes that are mechanistically distinct from, though metabolically intertwined with, proliferative reprogramming.

SLC27A1 does not appear to be significantly associated with lymph node metastasis or invasive behavior in most tumor types examined, suggesting that its primary oncological function is metabolic support for tumor growth rather than direct promotion of motility (Poenaru et al., 2025).

SLC27A2 plays a prominent pro-invasive role across multiple tumor types. In differentiated thyroid cancer, SLC27A2 is highly expressed in tumor tissues and cell lines and is closely associated with clinicopathological progression; its knockdown significantly inhibits proliferation and invasion through suppression of the MAPK pathway and downregulation of C-FOS (Zhu Y. et al., 2023). In endometrial cancer, the transcription factor FOXM1 directly activates SLC27A2 transcription, and elevated SLC27A2 expression positively correlates with poor prognosis, advanced pathological stage, and high clinical grade; silencing SLC27A2 markedly suppresses proliferation, migration, and metabolic activity in AN3CA and ISHIKAWA cell lines (Feng et al., 2018). In ccRCC, SLC27A2 expression is paradoxically reduced in tumor tissues relative to normal renal parenchyma, and its restoration inhibits tumor proliferation, migration, and invasion while improving patient prognosis (Xu et al., 2022)—a context in which SLC27A2 appears to act as a metastasis suppressor rather than a promoter. Furthermore, SLC27A2 negatively regulates EMT signaling by inhibiting CDK3; disruption of this SLC27A2–CDK3–EMT axis is considered a potential anti-metastatic therapeutic strategy (Xu et al., 2022).

SLC27A3 has been implicated in genomic instability-driven metastatic aggressiveness. In undifferentiated pleomorphic sarcoma (UPS), copy number gain of SLC27A3 (located at 1q21-q22) occurs in approximately 38% of cases and correlates with poor prognosis; genomic alterations involving SLC27A3 are more frequent in UPS than in leiomyosarcoma, suggesting that SLC27A3 amplification contributes to the enhanced aggressiveness characteristic of this sarcoma subtype (Silveira et al., 2013).

SLC27A4 drives invasion and metastasis in HCC through an epigenetically regulated non-coding RNA axis. The long non-coding RNA HOXD-AS1 acts as a competing endogenous RNA, sequestering miR-326 and thereby relieving miR-326-mediated suppression of SLC27A4, which in turn promotes HCC cell proliferation, invasion, and distant metastasis; disruption of this HOXD-AS1/miR-326/SLC27A4 regulatory axis represents a mechanistically important pathway in HCC progression (Ji et al., 2020).

SLC27A5 demonstrates tumor-type-specific behavior in the context of metastasis. Whereas SLC27A5 generally acts as a metastasis suppressor in HCC, its high expression in lung cancer is paradoxically associated with poor clinical outcomes (Liu et al., 2018), suggesting that the metabolic and microenvironmental context determines whether elevated SLC27A5 activity is protective or permissive for aggressive tumor behavior. This context-dependency highlights the need for tumor-type-specific interpretation of SLC27 family member expression profiles.

SLC27A6 promotes invasion and metastasis in papillary thyroid carcinoma (PTC), where it is highly expressed and positively correlated with N stage and BRAF mutation status. Knockdown of SLC27A6 inhibits PTC cell proliferation, migration, and invasion while inducing apoptosis, mediated at least in part through downregulation of c-MYC expression (Liu et al., 2022).

Overall, the SLC27 family contributes to metastatic progression through integrated regulation of lipid metabolism, EMT-related signaling, oncogenic transcription factor networks, and non-coding RNA axes. Individual members exhibit strikingly divergent—and in some cases opposing—effects depending on the tumor type and microenvironmental context, underscoring the complexity of SLC27-mediated metastasis regulation.

#### 1.4.3 Roles of the SLC27 family in drug resistance and therapeutic adaptation

Metabolic plasticity is a critical enabler of acquired therapeutic resistance. By dynamically remodeling fatty acid metabolism, redox homeostasis, and mitochondrial function, SLC27 family members enable tumor cells to survive chemotherapy, targeted agents, radiotherapy, and hormonal therapies. The resistance mechanisms mediated by individual SLC27 members are mechanistically diverse, spanning ferroptosis suppression, mitophagy induction, ABC transporter regulation, and lipid-mediated redox buffering.

SLC27A1 upregulation in various tumor contexts is associated with genomic instability and impaired immune surveillance, while paradoxically enhancing sensitivity to EGFR inhibitors such as gefitinib and erlotinib, suggesting that SLC27A1 shapes a metabolic state that differentially affects responses to distinct therapeutic modalities (Zhang W. et al., 2024). In DLBCL, co-overexpression of SLC27A1 with the nuclear receptor corepressor NCoR1 indicates increased reliance on oxidative metabolic pathways, which may selectively alter drug responsiveness in this lymphoma subtype (Magi et al., 2019).

SLC27A2 plays a multifaceted role in chemoresistance, primarily as a sensitizer whose downregulation confers resistance. In primary ovarian cancer, reduced SLC27A2 expression is associated with cisplatin resistance and poor prognosis; lentiviral restoration of SLC27A2 expression activates miR-411 transcription through direct promoter binding, suppresses the efflux transporter ABCG2, enhances apoptosis, and restores chemosensitivity (Chen et al., 2018). In cisplatin-resistant A2780 ovarian cancer cells, SLC27A2 expression is reduced by more than tenfold relative to sensitive parental cells (Januchowski et al., 2014). In CD166+ lung cancer stem cells, low SLC27A2 expression similarly correlates with cisplatin resistance; SLC27A2 upregulation enhances sensitivity by negatively regulating the Bmi1–ABCG2 axis (Su et al., 2016). Beyond platinum resistance, SLC27A2 promotes tumor proliferation through FAO regulation in prostate cancer; knockdown reduces tumor volume by approximately 35% (P < 0.05) and potentiates the cytotoxic effect of doxorubicin, supporting its role as a target for combination strategies (Tao et al., 2022).

SLC27A3 mediates resistance to targeted therapy through a mitochondrial stress–lipid storage circuit. As an acyl-CoA ligase, SLC27A3 generates long-chain fatty acyl-CoA species that fuel FAO; however, excessive FAO in the context of pazopanib treatment decreases mitochondrial membrane potential and elevates ROS, triggering PINK1/Parkin-dependent mitophagy. This adaptive mitophagy reduces ROS burden and concurrently suppresses CPT1A-dependent FAO, redirecting acyl-CoA toward triglyceride and cholesterol ester synthesis in lipid droplets and ultimately driving pazopanib resistance in clear cell RCC. Importantly, SLC27A3 knockdown reverses this resistance phenotype both *in vitro* and *in vivo*, validating it as a tractable therapeutic target in RCC (Lu D. et al., 2024).

SLC27A4 modulates targeted therapy sensitivity in HCC primarily through ferroptosis regulation. Its selective transport of MUFAs increases membrane MUFA content, reducing susceptibility to lipid peroxidation and ferroptosis; silencing of SLC27A4 significantly enhances sorafenib sensitivity in HCC cells by lowering the ferroptosis threshold (Li Z. et al., 2023).

SLC27A5 regulates drug sensitivity through multiple complementary mechanisms in HCC. Its deficiency leads to accumulation of polyunsaturated lipid species, elevating the NADP+/NADPH ratio and promoting ROS generation and lipid peroxidation; the resulting 4-HNE-mediated modification of KEAP1 activates the NRF2/TXNRD1 antioxidant pathway, promoting tumor cell survival and conferring sorafenib resistance—a vulnerability that can be exploited by pharmacological inhibition of TXNRD1 (Gao et al., 2020). In sorafenib-resistant HCC, SLC27A5 downregulation maintains glutathione homeostasis and suppresses ferroptosis through NRF2-dependent upregulation of glutathione reductase (GSR); combined treatment with GSR inhibitors such as carmustine effectively reverses resistance (Xu et al., 2023). SLC27A5 additionally counteracts radiotherapy resistance mediated by the E3 ubiquitin ligase UBAP2: UBAP2 degrades SLC27A5 *via* the ubiquitin-proteasome pathway to enhance RAD51-mediated homologous recombination repair and radioresistance, whereas SLC27A5 overexpression reverses this effect (Liu et al., 2024). These converging mechanisms position SLC27A5 as a multivalent regulator of therapeutic sensitivity across chemotherapy, targeted therapy, and radiotherapy.

SLC27A6 mediates resistance to androgen receptor-targeted therapy in prostate cancer. In enzalutamide-resistant prostate cancer models (LNCaP and C4-2B cell lines), SLC27A6 upregulation is associated with a metabolic shift characterized by increased lactate and citrate uptake, reduced glucose dependence, and enhanced lipid biosynthesis. Knockdown of SLC27A6 significantly suppresses proliferation and migration, induces G1/S cell cycle arrest and apoptosis (with BAX upregulation), and reduces the expression of Cyclin D1, CDK6, fatty acid synthase, and acetyl-CoA carboxylase, collectively reversing the lipogenic phenotype associated with enzalutamide resistance (Verma et al., 2020; Kushwaha et al., 2022).

Other members of the SLC superfamily also critically regulate ferroptosis and contribute to therapeutic adaptation, complementing the membrane lipid remodeling functions of SLC27 proteins. Notably, SLC7A11, the light chain of the cystine/glutamate antiporter, is frequently overexpressed in various cancers. By facilitating cystine uptake and subsequent glutathione biosynthesis, SLC7A11 effectively suppresses lipid peroxidation and ferroptosis, thereby enhancing tumor cell survival and conferring resistance to multiple therapies. This parallels the ferroptosis-modulatory roles of SLC27A4 and SLC27A5, further underscoring the importance of SLC transporters in mediating metabolic vulnerabilities and drug resistance.

#### 1.4.4 Roles of the SLC27 family in tumor immune regulation and immune escape

Lipid metabolism is increasingly recognized as a key determinant of immune cell function and tumor immune evasion. By controlling LCFA availability in the tumor microenvironment (TME), SLC27 family members can influence the metabolic fitness of infiltrating immune cells, alter cytokine signaling, and modulate the immunogenicity of tumor cells themselves—functions that extend the oncological significance of this family well beyond intrinsic tumor metabolism.

SLC27A1 participates in immune-metabolic crosstalk in the TME through its roles in oxidative metabolism and immune surveillance. Its upregulation is associated with impaired immune function at the tumor site (Zhang W. et al., 2024), and in DLBCL, co-overexpression of SLC27A1 and NCoR1 indicates a phenotype characterized by increased dependence on oxidative pathways, which may reshape immune cell interactions by altering local fatty acid availability (Magi et al., 2019). These observations suggest that SLC27A1-driven lipid uptake by tumor cells may competitively deprive immune effector cells of fatty acid substrates required for their activation and function, though the precise mechanisms warrant further investigation.

SLC27A2 displays heterogeneous, tumor-type-specific immune regulatory effects. In DLBCL, elevated SLC27A2 expression is positively associated with CD4^+^ T-cell, CD8^+^ T-cell, and macrophage infiltration, suggesting that in this context SLC27A2-driven lipid metabolic activity may support an immune-permissive microenvironment (Wang et al., 2024). By contrast, in ALL, high SLC27A2 expression is associated with poorer overall survival and event-free survival and negatively correlates with immune cell infiltration, implicating SLC27A2 in immune escape through the metabolic-immune axis (Lu L. et al., 2024). In ccRCC, where SLC27A2 is paradoxically downregulated in tumor tissues, restoration of SLC27A2 expression not only inhibits tumor proliferation, migration, and invasion but is also associated with improved patient prognosis (Xu et al., 2022), suggesting that SLC27A2 loss may create an immune-evasive lipid microenvironment in this cancer type.

SLC27A5 participates in immune regulation within the TME through its interconnected effects on lipid peroxidation, redox homeostasis, and EMT. In esophageal squamous cell carcinoma, SLC27A5 expression predicts sensitivity to neoadjuvant chemoradiotherapy, and low SLC27A5 expression is associated with poor prognosis (Guo et al., 2016). Beyond this prognostic value, SLC27A5 regulates EMT- and p53-related signaling pathways and influences immune cell distribution within the TME, indicating that SLC27A5-mediated metabolic states shape the immunological landscape of tumors (Cui et al., 2024). In HCC, SLC27A5 expression is closely linked to immunotherapy sensitivity in patients with high copper metabolism scores, and its upregulation of FDX1—a key cuproptosis regulator—may potentiate anti-tumor immune responses by promoting immunogenic tumor cell death (Li X. et al., 2023). These multifaceted immune-regulatory effects position SLC27A5 as a potentially important determinant of response to both conventional chemoradiotherapy and immune checkpoint-based therapies.

SLC27A2 and SLC27A4 have additionally been implicated in inflammatory bowel disease-associated carcinogenesis, where their upregulation in inflamed intestinal tissue promotes local lipid accumulation, lipid peroxidation, and DNA damage—a metabolic-inflammatory interface that may progressively erode immune tolerance and increase malignant transformation risk.Collectively, by controlling fatty acid flux in the TME, the SLC27 family shapes immune cell infiltration patterns, modulates the redox environment, influences EMT, and regulates tumor immunogenicity. These immune-metabolic interactions represent an emerging frontier in cancer biology and suggest that SLC27-targeted interventions may synergize with immunotherapy by simultaneously normalizing lipid metabolism and restoring immune competence in the TME.

Collectively, the SLC27 family fulfills multidimensional roles in cancer biology, with individual members contributing—often simultaneously—to metabolic reprogramming, invasion, drug resistance, and immune evasion. The oncological functions of SLC27A1, A2, A3, and A4 are predominantly pro-tumorigenic, while SLC27A5 primarily acts as a tumor suppressor in hepatic malignancies (with tumor-type-specific exceptions), and SLC27A6 exhibits context-dependent roles shaped by the normal-versus-malignant tissue status. This functional complexity, spanning multiple cancer hallmarks and tumor types, underscores both the challenge and the opportunity inherent in targeting the SLC27 family for precision oncological therapy.

### 1.5 Clinical value and translational significance of the SLC27 family in cancer

**FIGURE 4:**
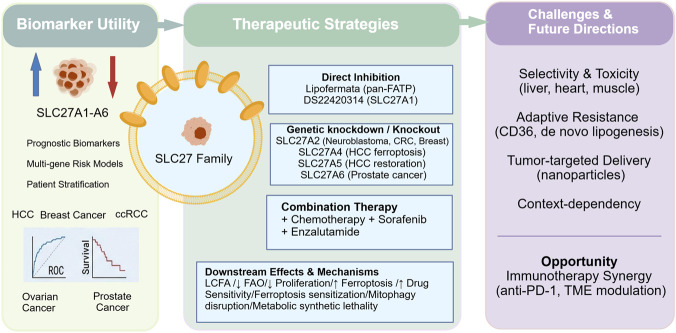
Therapeutic targeting strategies of the SLC27 family in cancer.

The mechanistic insights presented above establish the SLC27 family as a pervasive regulator of tumor biology. Translating this mechanistic knowledge into clinical utility requires a distinct analytical lens: rather than cataloguing individual molecular interactions, the following discussion asks which SLC27 members can serve as actionable biomarkers for patient stratification and prognosis, which represent tractable therapeutic targets, and what barriers currently impede clinical translation. Figure 4 summarizes current and potential strategies for therapeutically targeting the SLC27 family, including direct inhibition, genetic approaches, combination therapies, and their downstream effects on tumor biology.

#### 1.5.1 SLC27 family members as prognostic and diagnostic biomarkers

A defining feature of the SLC27 family in clinical oncology is the consistent, reproducible association between aberrant member expression and patient survival outcomes across diverse cancer types, a landscape comprehensively summarized in Table 2. What distinguishes the biomarker utility of these proteins is not merely their differential expression in tumors, but their independent prognostic power when integrated into multi-gene risk models—models that frequently outperform single-gene approaches and capture the metabolic heterogeneity of individual tumors.

SLC27A1 demonstrates prognostic value across multiple cancer lineages through its integration into composite gene signatures. In high-grade serous ovarian cancer, SLC27A1 forms part of a mitochondrial metabolism-related gene signature linked to copy number variation, and its expression is incorporated into risk score models that predict poor prognosis with validated discriminatory capacity (Meng et al., 2023). In lung adenocarcinoma, SLC27A1 is included in an 8-gene PPAR pathway-related prognostic model; high expression is associated with poor survival outcomes, a finding validated by receiver operating characteristic (ROC) curve analysis (Zhang W. et al., 2024). These multi-gene integrations suggest that SLC27A1’s prognostic value is best realized not in isolation but as a metabolic indicator within broader lipid-regulatory gene networks.

SLC27A2 exhibits one of the most clinically nuanced expression profiles within the family, with its prognostic direction depending critically on tumor type—a pattern that mirrors its context-dependent mechanistic roles described in Section 4. In ccRCC, high SLC27A2 expression is a favorable prognostic indicator for both disease-free survival (DFS) and overall survival (OS), and its incorporation into a nine-gene apoptosis-related prognostic model achieves an area under the ROC curve (AUC) exceeding 0.70, confirming independent prognostic capability (Cui et al., 2024). This favorable association likely reflects SLC27A2’s role as a metastasis suppressor in renal cancer, as discussed in Section 4.2. Conversely, in thyroid cancer, elevated SLC27A2 protein levels—quantified by immunohistochemistry in 98 patients—correlate with lymph node metastasis, advanced pathological stage, and worse clinical prognosis (Feng et al., 2022). In hematological malignancies, SLC27A2 upregulation in follicular lymphoma marks disease progression (Magi et al., 2019), while its downregulation in non-functional invasive pituitary tumors relative to non-invasive counterparts suggests it may serve as an indicator of tumor aggressiveness (Wang et al., 2019a). Across breast cancer, SLC27A2 cytoplasmic expression is detectable in approximately 30% of long-term survivors and 20% of short-term survivors, though without reaching statistical significance for disease-specific survival in a cohort of 80 patients (Shubbar et al., 2013)—indicating that the clinical translation of SLC27A2 as a breast cancer biomarker requires further validation in larger, prospectively designed cohorts. Notably, through CRISPR-based functional screening, SLC27A2 has been identified as a regulatory target of the fluorescent probe cLG, which—acting in synergy with sphingomyelin phosphodiesterase 1 (SMPD1)—enables complementary optical imaging differentiation between HCC and adjacent normal liver tissue, a proof-of-concept application with direct relevance to fluorescence-guided surgical resection (Wang et al., 2021).

SLC27A3 has emerging biomarker value in lipid-rich and aggressive tumor contexts, though its clinical validation lags behind other family members. In ccRCC, high SLC27A3 transcript levels are associated with shorter OS, disease-specific survival (DSS), and progression-free survival (PFS), positioning it as a potential unfavorable prognostic indicator in this cancer type (Lu D. et al., 2024). In pleomorphic sarcomas and leiomyosarcomas, quantitative transcript analysis confirms significantly elevated SLC27A3 expression, and its copy number gain in UPS provides a genomic basis for predicting aggressive tumor behavior (Silveira et al., 2013). In colorectal cancer, SLC27A3 transcripts are upregulated in tumor *versus* adjacent normal tissue, though the independent prognostic significance of this finding requires further characterization (Niculae et al., 2022).

SLC27A4 demonstrates the broadest and most consistently adverse prognostic associations across solid tumors, making it one of the most clinically relevant members of the family for biomarker development. In breast cancer, high SLC27A4 expression correlates with poor OS and distant metastasis-free survival (DMFS), as validated by immunohistochemical and bioinformatic analyses (Yen et al., 2018). In ccRCC, membranous SLC27A4 overexpression—detected by IHC in 180 patients—is associated with high tumor grade, advanced stage, and increased recurrence rates, with high staining intensity independently predicting recurrence-free survival (Kim et al., 2019). In bladder cancer, SLC27A4 overexpression is found in over 47% of tumor specimens and associates with high grade, advanced stage, and poor OS in a cohort of 286 patients (Jeong et al., 2021). In HCC, elevated SLC27A4 transcript levels independently predict shorter OS and recurrence-free survival (Li Z. et al., 2023; Yeung et al., 2025). In ovarian cancer, significant overexpression of SLC27A4 is linked to poorer OS and relapse-free survival, particularly in fatty acid-enriched microenvironments (Chen et al., 2021; Gao et al., 2024). In lung adenocarcinoma, SLC27A4 is incorporated into a multi-SLC-transporter prognostic risk score model that effectively stratifies patients by survival probability (Zheng et al., 2023). In colorectal cancer, although SLC27A4 transcript levels are paradoxically reduced in tumor *versus* adjacent normal tissue (Niculae et al., 2022), high SLC27A4 expression is confirmed as an independent adverse prognostic factor by both univariate and multivariate Cox regression analyses, and it contributes to an 11-gene lipid metabolism prognostic model—including CPT1C—with high predictive efficiency for survival and clinicopathological characteristics (Yang et al., 2024). This apparent discrepancy between expression direction and prognostic impact across colorectal cancer studies highlights the importance of study methodology (transcript vs. protein quantification, tumor-versus-normal comparisons vs. within-tumor stratification) and warrants harmonized multi-cohort validation.

SLC27A5 occupies a distinctive clinical position as a predominantly downregulated, tumor-suppressive biomarker whose low expression consistently signals poor prognosis—particularly in liver cancer. In HCC, IHC analysis of 40 tumor specimens confirms that reduced SLC27A5 levels are associated with poor differentiation, early recurrence, and adverse clinical outcomes; transcriptomic analysis across larger datasets corroborates that high SLC27A5 expression predicts favorable OS and DFS (Gao et al., 2020; Zhang F. et al., 2021). In a clinically applicable PPAR-related multigene prognostic scoring system for HCC constructed from SLC27A5, MMP1, and HMGCS2, low SLC27A5 expression contributes to a high-risk score (risk formula: 0.1488 × MMP1 − 0.0393 × HMGCS2 − 0.0479 × SLC27A5; HR = 2.72, P < 0.001) (Xu et al., 2021), demonstrating its additive value within composite metabolic signatures. In breast cancer, SLC27A5 expression is significantly associated with disease-free survival and risk of distant metastasis in transcriptomic analyses (Xu et al., 2025), extending its potential biomarker utility beyond liver malignancy. In esophageal cancer, SLC27A5 expression predicts not only prognosis but also patient responsiveness to oxaliplatin-based chemotherapy (Cui et al., 2024), positioning it as a dual prognostic-predictive biomarker with potential to guide treatment selection. In esophageal squamous cell carcinoma, a multi-dimensional signature combining protein-coding genes and long non-coding RNAs—including SLC27A5—achieves significant prognostic stratification (Guo et al., 2016). In lung cancer, high SLC27A5 expression is paradoxically associated with poor OS (Liu et al., 2018), underscoring once again that its clinical interpretation is inherently tumor-type-dependent and cannot be extrapolated across cancer lineages without validation.

SLC27A6 exhibits cancer-type-specific expression alterations with emerging prognostic and predictive relevance. In prostate cancer, high SLC27A6 expression correlates with poorer survival outcomes and is mechanistically linked to enzalutamide resistance, positioning it as both a prognostic and predictive biomarker for treatment responsiveness (Verma et al., 2020; Kushwaha et al., 2022). In PTC, quantitative proteomic analysis reveals upregulated SLC27A6 expression that is negatively correlated with a panel of downregulated proteins (including HGD, CA4, COL23A1, SLC26A7, FHL1, and TPO), forming a protein interaction network predictive of tumor invasiveness (Dai et al., 2020). In ovarian cancer, IHC of 120 specimens demonstrates significant SLC27A6 downregulation in tumor tissues with corresponding associations with poor OS (Chen et al., 2021). In colorectal cancer, a discordance between reduced transcript and preserved protein expression levels highlights the importance of post-transcriptional regulation in SLC27A6 biology and complicates its straightforward deployment as a tissue biomarker (Gao et al., 2025).

Taken together, the SLC27 family members exhibit clearly defined, cancer-type-specific expression abnormalities with demonstrable clinical prognostic relevance. Their greatest biomarker utility emerges not from single-marker applications but from integration into multi-gene metabolic signatures that capture the compositional complexity of tumor lipid dependency.

#### 1.5.2 SLC27 family members as therapeutic targets

Beyond their value as biomarkers, the functional dependence of certain tumor types on SLC27-mediated lipid import creates discrete metabolic vulnerabilities that can be pharmacologically exploited. The therapeutic strategies under development span direct enzyme inhibition, combination regimens that exploit metabolic synthetic lethality, and novel delivery approaches.

The most advanced therapeutic evidence involves SLC27A1 and SLC27A2 inhibition using Lipofermata, a pan-FATP small-molecule inhibitor. In melanoma, Lipofermata significantly reduces tumor growth and invasion by disrupting SLC27A1-mediated lipid transfer from peritumoral adipocytes to tumor cells—a mechanism of particular relevance in adipose-rich tumor microenvironments (Zhang et al., 2018). In breast cancer, Lipofermata suppresses LCFA uptake and reduces tumor growth in SLC27A2-dependent cell lines (Zhang B. et al., 2024), and in prostate cancer, SLC27A2 knockdown reduces tumor volume by approximately 35% (P < 0.05) and potentiates the cytotoxic effect of doxorubicin, supporting its utility in combination regimens (Tao et al., 2022). The more selective inhibitor DS22420314—an arylpiperazine compound targeting SLC27A1—effectively suppresses LCFA uptake and cell viability in breast cancer models, offering improved specificity over pan-FATP approaches (Mendes et al., 2019).

SLC27A2 represents perhaps the most extensively validated therapeutic target within the family, with preclinical evidence across multiple tumor types supporting both monotherapy and combination strategies. In MYCN-amplified neuroblastoma, pharmacological inhibition of SLC27A2 selectively suppresses tumor growth, prolongs animal survival, and synergizes with conventional chemotherapy in multiple preclinical models (Tao et al., 2022). In CRC, both genetic deletion and pharmacological inhibition of SLC27A2 significantly reduce tumor growth and extend survival in mouse models, with enhanced antitumor effects observed when combined with standard chemotherapy; this therapeutic effect is mechanistically linked to disruption of PPAR-mediated lipid metabolic reprogramming (Shang et al., 2023). In breast cancer, a four-gene nucleotide metabolism-related risk model positions SLC27A2 as an actionable target whose inhibition by Lipofermata significantly suppresses tumor growth in cell lines (Zhang B. et al., 2024). In ccRCC, SLC27A2 restoration suppresses tumor proliferation, migration, and invasion while antagonizing the CDK3–EMT axis—a non-lipid-transport function that broadens its therapeutic rationale (Xu et al., 2022). The ability of SLC27A2 to convert small-molecule inhibitors of ATP citrate lyase (ACLY) and exert indirect anticancer functions through metabolic pathway crosstalk further expands its therapeutic interface (Gautam et al., 2025).

SLC27A3 knockdown reverses pazopanib resistance in ccRCC through disruption of the PINK1/Parkin mitophagy circuit and redirection of acyl-CoA away from FAO toward lipid droplet storage, validating it as a target for overcoming targeted therapy resistance in renal cancer (Lu D. et al., 2024).

SLC27A4 and SLC27A5 emerge as critical targets for modulating ferroptosis sensitivity in HCC. Silencing SLC27A4 lowers the ferroptosis threshold by reducing MUFA membrane incorporation, significantly enhancing sorafenib responsiveness (Li Z. et al., 2023). Restoration or pharmacological support of SLC27A5 function in HCC sensitizes cells to sorafenib by promoting lipid peroxidation, activating the NRF2/TXNRD1 pathway, and maintaining glutathione homeostasis through GSR (Gao et al., 2020; Xu et al., 2023). Combined HNF4A agonist and AKT inhibitor therapy exploits the hypoxia-induced SLC27A5 downregulation axis to synergistically suppress HCC tumor growth (Tao et al., 2025), illustrating how upstream regulators of SLC27A5 expression can themselves be therapeutic entry points.

SLC27A6 represents an emerging therapeutic target in endocrine therapy-resistant prostate cancer, where its knockdown reverses the metabolic phenotype of enzalutamide resistance—including reduction of lipogenic enzyme expression and restoration of glucose-dependent metabolism (Verma et al., 2020; Kushwaha et al., 2022)—and in PTC, where SLC27A6 silencing reduces proliferation and invasion through c-MYC downregulation (Liu et al., 2022).

#### 1.5.3 Challenges and future directions in clinical translation

Despite the compelling preclinical evidence outlined above, several critical barriers currently limit the clinical translation of SLC27-targeted strategies, and their honest appraisal is essential for setting realistic research priorities.

Selectivity and on-target toxicity represent the foremost concern. The SLC27 family is broadly expressed in metabolically active normal tissues—liver, skeletal muscle, heart, and adipose tissue—and the physiological functions of its members, particularly in hepatic lipid handling and cardiac energy supply (as detailed in Section 3), create a narrow therapeutic window for systemic inhibition. Inhibition of SLC27A2 in normal hepatocytes and adipocytes risks inducing hepatic steatosis and metabolic dysregulation, necessitating tumor-targeted delivery strategies that spare normal tissue. Lipofermata’s pan-FATP activity further exacerbates this selectivity problem, and more isoform-selective inhibitors with defined structure-activity relationships are urgently needed.

Adaptive resistance poses a second major challenge. Tumor cells subjected to SLC27 inhibition may upregulate compensatory fatty acid transporters—including CD36, FABP family members, or other SLC27 family members—or shift their metabolic dependency toward *de novo* lipogenesis or glucose oxidation, thereby bypassing the therapeutic blockade. The mitophagy-mediated pazopanib resistance driven by SLC27A3 (Lu D. et al., 2024) exemplifies the capacity of tumor cells to rewire metabolic networks around single-transporter blockades. Combination strategies that simultaneously target complementary lipid uptake pathways or couple SLC27 inhibition with suppression of *de novo* lipogenesis (e.g., FASN or ACLY inhibitors) may be required to achieve durable antitumor responses.

Drug delivery and tumor penetration constitute a third barrier. Given that SLC27 proteins are integral membrane proteins, small-molecule inhibitors must achieve sufficient membrane permeation and intratumoral accumulation to engage their targets effectively. Encapsulation in lipid nanoparticles or tumor-targeting delivery platforms may improve tissue selectivity, though the interaction of lipid-based carriers with LCFA transport mechanisms requires careful characterization.

Context-dependency of expression adds interpretive complexity to biomarker-guided patient selection. As repeatedly illustrated above—particularly for SLC27A5 and SLC27A6—the same family member may function as an oncogene in one tumor type and a tumor suppressor in another. This precludes pan-cancer biomarker deployment and demands tumor-type-specific clinical validation frameworks. Single-cell RNA sequencing and spatial transcriptomics will be essential tools for resolving intra-tumoral SLC27 expression heterogeneity and identifying the specific tumor cell populations in which SLC27-targeted interventions are most likely to be effective.

Integration with immunotherapy represents a frontier opportunity. Given the emerging evidence that SLC27 members shape immune cell infiltration patterns, tumor immunogenicity, and checkpoint sensitivity—most notably through SLC27A5’s influence on cuproptosis, ferroptosis, and the TME immune landscape—future clinical trials should be designed to prospectively evaluate SLC27 expression as a predictive biomarker for immune checkpoint inhibitor response, and to assess whether SLC27-targeted metabolic normalization can enhance the efficacy of anti-PD-1/PD-L1 therapies.

In conclusion, the SLC27 family stands at a promising but incompletely realized frontier of metabolic oncology. Its members offer dual utility as clinically informative biomarkers and mechanistically validated therapeutic targets, with the most actionable evidence concentrated around SLC27A1, A2, A4, and A5 in specific tumor contexts. Realizing the full clinical potential of this protein family will require the development of isoform-selective inhibitors, tumor-targeted delivery systems, multi-omics patient stratification frameworks, and combination regimens designed to preempt metabolic adaptation. These translational priorities, if addressed systematically, position the SLC27 family as a tractable node for precision oncological intervention in metabolically dependent cancers.

## 2 Summary and outlook

The SLC27 family, as long-chain fatty acid membrane transporters, plays a multi-level and multi-dimensional role in the initiation, progression, and treatment response of human cancers. Its involvement in metabolic reprogramming, immune regulation, and drug resistance mechanisms within the tumor microenvironment has been increasingly validated by clinical studies, highlighting its critical position in cancer research. With the rapid development of multi-omics technologies, continuous optimization of targeted drugs, and advancing clinical trials, the molecular mechanisms related to the SLC27 family will become clearer, and its application prospects in precision oncology will gradually be realized. In the future, by optimizing drug delivery systems, overcoming drug resistance challenges, and exploring personalized treatment strategies, the SLC27 family is expected to provide new breakthroughs for early cancer diagnosis and precision therapy, ultimately improving patient survival quality and clinical outcomes.

## References

[B1] Acharya N. K. Levin E. C. Clifford P. M. Han M. Nagele R. G. Gold L. I. (2023). Dysregulation of fatty acid transport protein 2 (FATP2/SLC27A2) in adipocyte-derived lipids mediate melanoma progression *via* FATP proteins.neurodegenerative diseases: implications for lipid metabolism and neuroinflammation. J. Neurochem. 164 (4), 512–525. 10.1111/jnc.15723 36437609

[B2] Akhouri V. Majumder S. Gaikwad A. B. (2023). Targeting DNA methylation in diabetic kidney disease: a new perspective. Life Sci. 335, 122256. 10.1016/j.lfs.2023.122256 37949210

[B3] Akiyama M. Ishigaki K. Sakaue S. Momozawa Y. Horikoshi M. Hirata M. (2019). Characterizing rare and low-frequency height-associated variants in the Japanese population. Nat. Commun. 10 (1), 4393. 10.1038/s41467-019-12276-5 31562340 PMC6764965

[B4] AmeliMojarad M. AmeliMojarad M. Cui X. (2024). Discovering the lipid metabolism-related hub genes of HCC-treated samples with PPARα agonist through weighted correlation network analysis. Sci. Rep. 14 (1), 19591. 10.1038/s41598-024-69998-w 39179766 PMC11344068

[B5] Anderson C. M. Stahl A. (2013). SLC27 fatty acid transport proteins. Mol. Aspects Medicine 34 (2-3), 516–528. 10.1016/j.mam.2012.07.010 23506886 PMC3602789

[B6] Ason B. Castro-Perez J. M. Tep S. Stefanni A. Tadin-Strapps M. Hubbard B. K. (2011). ApoB knockdown in mice with antisense oligonucleotides reveals a surprising role for FATP5 in bile acid metabolism but not triglyceride accumulation. J. Lipid Res. 52 (6), 1146–1155. 10.1194/jlr.M013748

[B7] Auinger A. Helwig U. Pfeuffer M. Rubin D. Luedde M. Rausche T. (2012). A variant in the heart-specific fatty acid transport protein 6 is associated with lower fasting and postprandial TAG, blood pressure and left ventricular hypertrophy. Br. Journal Nutrition 107 (10), 1422–1428. 10.1017/S0007114511004727 21920065

[B8] Benninghoff T. Espelage L. Eickelschulte S. Zeinert I. Sinowenka I. Müller F. (2020). The RabGAPs TBC1D1 and TBC1D4 control uptake of long-chain fatty acids into skeletal muscle *via* fatty Acid transporter SLC27A4/FATP4. Diabetes 69 (11), 2281–2293. 10.2337/db20-0180 32868338

[B9] Blitek A. Szymanska M. (2024). Expression profiles of Fatty acid transporters and the role of n-3 and n-6 polyunsaturated Fatty acids in the porcine endometrium. Int. J. Mol. Sci. 25 (20), 11102. 10.3390/ijms252011102 39456882 PMC11507490

[B10] Castro-Perez J. M. Roddy T. P. Shah V. Wang S. P. Ouyang X. Ogawa A. (2011). Attenuation of Slc27a5 gene expression followed by LC-MS measurement of bile acid reconjugation using metabolomics and a stable isotope tracer strategy. J. Proteome Res. 10 (10), 4683–4691. 10.1021/pr200475g 21819150

[B11] Chabowski A. Żendzian-Piotrowska M. Konstantynowicz K. Pankiewicz W. Mikłosz A. Łukaszuk B. (2013). Fatty acid transporters involved in the palmitate and oleate induced insulin resistance in primary rat hepatocytes. Acta Physiol. 207 (2), 346–357. 10.1111/apha.12022 23140342

[B12] Chakraborty P. Niewiadomska M. Farhat K. Morris L. Whyte S. Humphries K. M. (2024). Effect of low-level tragus stimulation on cardiac metabolism in heart failure with preserved ejection fraction: a transcriptomics-based analysis. Int. J. Mol. Sci. 25 (8), 4312. 10.3390/ijms25084312 38673896 PMC11050145

[B13] Challa T. D. Straub L. G. Balaz M. Kiehlmann E. Donze O. Rudofsky G. (2015). Regulation of *de novo* Adipocyte Differentiation Through Cross Talk Between Adipocytes and Preadipocytes. Diabetes 64 (12), 4075–4087. 10.2337/db14-1932 26340931

[B14] Chen F. D. Chen H. H. Ke S. C. Zheng L. R. Zheng X. Y. (2018). SLC27A2 regulates miR-411 to affect chemo-resistance in ovarian cancer. Neoplasma 65 (6), 915–924. 10.4149/neo_2018_180122N48 30334452

[B15] Chen L. Vasoya R. P. Toke N. H. Parthasarathy A. Luo S. Chiles E. (2020). HNF4 regulates fatty acid oxidation and is required for renewal of intestinal stem cells in mice. Gastroenterology 158 (4), 985–999.e9. 10.1053/j.gastro.2019.11.031 31759926 PMC7062567

[B16] Chen H. Han T. Zhao Y. Feng L. (2021). Identification of solute-carrier family 27A molecules (SCL27As) as a potential biomarker of ovarian cancer based on bioinformatics and experiments. Ann. Transl. Med. 9 (15), 1237. 10.21037/atm-21-3026 34532374 PMC8421936

[B17] Cheng J. Sun R. Nie D. Li B. Gui S. B. Li C. Z. (2022). Identification and verification of SLC27A1, PTBP1 and EIF5A with significantly altered expression in aggressive pituitary adenomas. Front. Surgery 9, 923143. 10.3389/fsurg.2022.923143 PMC9275011 35836612

[B18] Chiu H. C. Kovacs A. Blanton R. M. Han X. Courtois M. Weinheimer C. J. (2005). Transgenic expression of fatty acid transport protein 1 in the heart causes lipotoxic cardiomyopathy. Circul. Res. 96 (2), 225–233. 10.1161/01.RES.0000154079.20681.B9 15618539

[B19] Chiu H. C. Kovacs A. Blanton R. M. Han X. Courtois M. Weinheimer C. J. (2023). Overexpression of fatty acid transport protein 1 (SLC27A1/FATP1) in skeletal muscle and adipose tissue exacerbates insulin resistance in obesity. Diabetes 72 (5), 645–656. 10.2337/db22-0876

[B20] Cui Y. Wen J. Fu J. Leng C. (2024). Identification of key genes to predict response to chemoradiotherapy and prognosis in esophageal squamous cell carcinoma. Front. Mol. Biosci. 11, 1512715. 10.3389/fmolb.2024.1512715 39633985 PMC11614722

[B21] Currie E. Schulze A. Zechner R. Walther T. C. Farese R. V. Jr. (2013). Cellular fatty acid metabolism and cancer. Cell Metab. 18 (2), 153–161. 10.1016/j.cmet.2013.05.017 23791484 PMC3742569

[B22] Dai J. Yu X. Han Y. Chai L. Liao Y. Zhong P. (2020). TMT-labeling proteomics of papillary thyroid carcinoma reveal invasive biomarkers. J. Cancer 11 (20), 6122–6132. 10.7150/jca.47290 32922552 PMC7477402

[B23] Digel M. Staffer S. Ehehalt F. Stremmel W. Ehehalt R. Füllekrug J. (2011). FATP4 contributes as an enzyme to the basal and insulin-mediated fatty acid uptake of C_2_C_12_ muscle cells. Am. J, Physiol. Endocrinol. Metabolism 301 (5), E785–E796. 10.1152/ajpendo.00079.2011 21750264

[B24] Doege H. Baillie R. A. Ortegon A. M. Tsang B. Wu Q. Punreddy S. (2006). Targeted deletion of FATP5 reveals multiple functions in liver metabolism: alterations in hepatic lipid homeostasis. Gastroenterology 130 (4), 1245–1258. 10.1053/j.gastro.2006.02.006 16618416

[B25] Ellis J. M. Li L. O. Wu P. Koves T. R. Ilkayeva O. Stevens R. D. (2010). Adipose acyl-CoA synthetase-1 directs fatty acids toward β-oxidation and is required for cold thermogenesis. Cell Metab. 12 (1), 53–64. 10.1016/j.cmet.2010.05.012 20620995 PMC2910420

[B26] Falany C. N. Xie X. Wheeler J. B. Wang J. Smith D. He D. (2002). Molecular cloning and expression of rat liver bile acid CoA ligase. J. Lipid Res. 43 (12), 2062–2070. 10.1194/jlr.M200254-JLR200 12454267

[B27] Falcon A. Doege H. Fluitt A. Tsang B. Watson N. Kay M. A. (2010). FATP2 is a hepatic fatty acid transporter and peroxisomal very long-chain acyl-CoA synthetase. Am. J. Physiol. Endocrinol. Metabolism 299 (3), E384–E393. 10.1152/ajpendo.00226.2010 20530735 PMC2944282

[B28] Feng Y. Li S. Zhang R. Liu F. Xu Q. Ding H. (2018). FOXM1 as a prognostic biomarker promotes endometrial cancer progression *via* transactivation of SLC27A2 expression. Int. J. Clin. Exp. Pathol. 11 (8), 3846–3857. 31949772 PMC6962789

[B29] Feng K. Ma R. Li H. Yin K. Du G. Chen X. (2022). Upregulated SLC27A2/FATP2 in differentiated thyroid carcinoma promotes tumor proliferation and migration. J. Clin. Lab. Anal. 36 (1), e24148. 10.1002/jcla.24148 34854499 PMC8761402

[B30] Gao Q. Zhang G. Zheng Y. Yang Y. Chen C. Xia J. (2020). SLC27A5 deficiency activates NRF2/TXNRD1 pathway by increased lipid peroxidation in HCC. Cell Death Differ. 27 (3), 1086–1104. 10.1038/s41418-019-0399-1 31367013 PMC7206086

[B31] Gao M. Lee S. H. Kwon H. Y. Ciaramicoli L. M. Jo E. Yu Y. H. (2024). A pair of fluorescent probes enabling precise diagnosis of liver cancer by complementary imaging. ACS Central Sci. 11 (1), 76–83. 10.1021/acscentsci.4c01822 PMC11758269 39866701

[B32] Gao B. Hu J. Wu H. Li B. (2025). Identification of lipid metabolism-related marker genes in colorectal cancer. Am. J. Cancer Res. 15 (5), 2022–2040. 10.62347/EGUX7327 40520872 PMC12163457

[B87] Gautam J. Wu J. Lally J. S. V. McNicol J. D. Fayyazi R. Ahmadi E. (2025). ACLY inhibition promotes tumour immunity and suppresses liver cancer. Nature 645 (8080), 507–517. 10.1038/s41586-025-09297-0 40739358 PMC12422966

[B33] Gillberg L. Perfilyev A. Brøns C. Thomasen M. Grunnet L. G. Volkov P. (2016). Adipose tissue transcriptomics and epigenomics in low birthweight men and controls: role of high-fat overfeeding. Diabetologia 59 (4), 799–812. 10.1007/s00125-015-3852-9 26750116

[B34] Gimeno R. E. Ortegon A. M. Patel S. Punreddy S. Ge P. Sun Y. (2003). Characterization of a heart-specific fatty acid transport protein. J. Biol. Chem. 278 (18), 16039–16044. 10.1074/jbc.M211412200 12556534

[B35] Gordaliza-Alaguero I. Sànchez-Fernàndez-de-Landa P. Radivojevikj D. Villarreal L. Arauz–Garofalo G. Gay M. (2025). Endogenous interactomes of MFN1 and MFN2 provide novel insights into interorganelle communication and autophagy. Autophagy 21 (5), 957–978. 10.1080/15548627.2024.2440843 39675054 PMC12013434

[B36] Grommes C. Landreth G. E. Heneka M. T. (2004). Antineoplastic effects of peroxisome proliferator-activated receptor gamma agonists. Lancet. Oncol. 5 (7), 419–429. 10.1016/S1470-2045(04)01509-8 15231248

[B37] Guo J. C. Li C. Q. Wang Q. Y. Zhao J. M. Ding J. Y. Li E. M. (2016). Protein-coding genes combined with long non-coding RNAs predict prognosis in esophageal squamous cell carcinoma patients as a novel clinical multi-dimensional signature. Mol. Biosyst. 12 (11), 3467–3477. 10.1039/c6mb00585c 27714034

[B38] Heather L. C. Cole M. A. Lygate C. A. Evans R. D. Stuckey D. J. Neubauer S. (2006a). Fatty acid transporter levels are preserved in the rat heart following a myocardial infarction despite reductions in fatty acid oxidation. J. Mol. Cell. Cardiol. 40 (6), 975–976. 10.1016/j.yjmcc.2006.03.423

[B39] Heather L. C. Cole M. A. Lygate C. A. Evans R. Stuckey D. J. Neubauer S. (2006b). Fatty acid transporter levels and palmitate oxidation rate correlate with ejection fraction in the infarcted rat heart. Cardiovasc. Res. 70 (3), 430–439. 10.1016/j.cardiores.2006.02.020 17034771

[B40] Hermoso M. A. Saez F. Villaverde C. Roldan E. R. S. (2020). Epididymal clear cells and their role in sperm maturation: expression of lipid metabolism genes and interaction with epididymosomes. Biol. Reproduction 103 (5), 987–999. 10.1093/biolre/ioaa134

[B41] Hirschmugl B. Desoye G. Catalano P. Klymiuk I. Scharnagl H. Payr S. (2017). Maternal obesity modulates intracellular lipid turnover in the human term placenta. Int. J. Obes. 41 (2), 317–323. 10.1038/ijo.2016.188 27780978 PMC5309341

[B42] Holloway G. P. Chou C. J. Lally J. Stellingwerff T. Maher A. C. Gavrilova O. (2011). Increasing skeletal muscle fatty acid transport protein 1 (SLC27A1/FATP1) targets fatty acids to oxidation and does not predispose mice to diet-induced insulin resistance. Diabetologia 54 (6), 1457–1467. 10.1007/s00125-011-2114-8 21442160

[B43] Hubbard B. Doege H. Punreddy S. Wu H. Huang X. Kaestner K. H. (2006). Mice deleted for fatty acid transport protein 5 have defective bile acid conjugation and are protected from obesity. J. Biol. Chem. 281 (40), 30653–30661. 10.1074/jbc.M606473200 16618417

[B44] Hui T. Y. Frohnert B. I. Smith A. J. Schaffer J. E. Bernlohr D. A. (1998). Molecular mechanisms of insulin-mediated regulation of fatty acid transport protein (FATP) expression in adipocytes. J. Biol. Chem. 273 (42), 27420–27429. 10.1074/jbc.273.42.27420 9765271

[B45] Januchowski R. Zawierucha P. Ruciński M. Andrzejewska M. Wojtowicz K. Nowicki M. (2014). Drug transporter expression profiling in chemoresistant variants of the A2780 ovarian cancer cell line. Biomed. Pharmacother. 68 (4), 447–453. 10.1016/j.biopha.2014.02.002 24814220

[B46] Jeong H. Oh H. E. Kim H. Lee J. H. Lee E. S. Kim Y. S. (2021). Upregulation of Fatty acid transporters is associated with tumor progression in non-muscle-invasive bladder cancer. Pathol. Oncol. Res. 27, 594705. 10.3389/pore.2021.594705 34257543 PMC8262182

[B47] Ji W. Wang Q. Yang J. (2020). LncRNA HOXD-AS1 promotes the metastasis of human hepatocellular carcinoma *via* modulating miR-326/SLC27A4. Cancer Cell Internat. 20, 161. 10.1186/s12935-020-01217-8 32425696 PMC7216491

[B48] Jia Z. Moulson C. L. Pei Z. Miner J. H. Watkins P. A. (2007). Fatty acid transport protein 4 is the principal very long chain fatty acyl-CoA synthetase in skin fibroblasts. J. Biol. Chem. 282 (28), 20573–20583. 10.1074/jbc.M700568200 17522045

[B49] Kazantzis M. Stahl A. (2011). Fatty acid transport proteins, implications in physiology and disease. Biochim. Biophys. Acta (BBA) - Mol. Cell Biol. Lipids 1821 (5), 852–857. 10.1016/j.bbalip.2011.09.010 21979150 PMC3274620

[B50] Khan S. Cabral P. D. Schilling W. P. Schmidt Z. W. Uddin A. N. Gingras A. (2018). Kidney proximal tubule lipoapoptosis is regulated by Fatty acid Transporter-2 (FATP2). J. Am. Soc. Nephrol. 29 (1), 81–91. 10.1681/ASN.2017030314 28993506 PMC5748912

[B51] Khan S. Gaivin R. Abramovich C. Boylan M. Calles J. Schelling J. R. (2020a). Fatty acid transport Protein-2 regulates glycemic control and diabetic kidney disease progression. JCI Insight 5 (15), e136845. 10.1172/jci.insight.136845 32614804 PMC7455077

[B52] Khan S. Cabral P. D. Schilling W. P. Schmidt M. (2020b). Fatty acid transport protein 3 (FATP3) exacerbates lipotoxicity and glucolipotoxicity in diabetic nephropathy by modulating long-chain fatty acid uptake in proximal tubules. Kidney Int. 97 (5), 912–923. 10.1016/j.kint.2019.12.015

[B53] Kim J. K. Gimeno R. E. Higashimori T. Kim H. J. Choi H. Punreddy S. (2004). Inactivation of fatty acid transport protein 1 prevents fat-induced insulin resistance in skeletal muscle. J. Clin. Invest. 113 (5), 756–763. 10.1172/JCI18917 14991074 PMC351314

[B54] Kim Y. S. Jung J. Jeong H. Lee J. H. Oh H. E. Lee E. S. (2019). High membranous expression of Fatty acid transport protein 4 is associated with tumorigenesis and tumor progression in clear cell renal cell carcinoma. Dis. Markers 2019, 5702026. 10.1155/2019/5702026 31089396 PMC6476224

[B55] Klar J. Schweiger M. Zimmerman R. Zechner R. Li H. Törmä H. (2009). Mutations in the fatty acid transport protein 4 gene cause ichthyosis prematurity syndrome. Am. J. Hum. Genet. 85 (2), 248–253. 10.1016/j.ajhg.2009.06.021 19631310 PMC2725242

[B56] Korbecki J. Kojder K. Jeżewski D. Simińska D. Tomasiak P. Tarnowski M. (2023). Reduced expression of very-long-chain Acyl-CoA synthetases *SLC27A4* and *SLC27A6* in the glioblastoma tumor compared to the peritumoral area. Brain Sci. 13 (5), 771. 10.3390/brainsci13050771 37239243 PMC10216168

[B57] Koundouros N. Poulogiannis G. (2020). Reprogramming of fatty acid metabolism in cancer. Br. J. Cancer 122 (1), 4–22. 10.1038/s41416-019-0650-z 31819192 PMC6964678

[B58] Kushwaha P. P. Verma S. S. Shankar E. Lin S. Gupta S. (2022). Role of solute carrier transporters SLC25A17 and SLC27A6 in acquired resistance to enzalutamide in castration-resistant prostate cancer. Mol. Carcinogenesis 61 (4), 397–407. 10.1002/mc.23383 34939235

[B59] Lee Y. T. Sun N. Kim M. Wang J. J. Tran B. V. Zhang R. Y. (2022). Circulating Tumor cell-based messenger RNA scoring System for prognostication of hepatocellular carcinoma: translating tissue-based messenger RNA profiling into a noninvasive setting. Liver Transpl. 28 (2), 200–214. 10.1002/lt.26337 34664394 PMC8820407

[B60] Lemberger L. Wagner R. Heller G. Pils D. Grunt T. W. (2022). Pharmacological inhibition of lipid import and transport proteins in ovarian cancer. Cancers 14 (23), 6004. 10.3390/cancers14236004 36497485 PMC9737127

[B61] Li S. Lee J. Zhou Y. Gordon W. C. Hill J. M. Bazan N. G. (2013a). Fatty acid transport protein 4 (FATP4) prevents light-induced degeneration of cone and rod photoreceptors by inhibiting RPE65 isomerase. J. Neurosci. 33 (7), 3178–3189. 10.1523/JNEUROSCI.2428-12.2013 23407971 PMC3625017

[B62] Li J. Zhang Y. Zhou L. (2013b). Genetic polymorphisms in SLC27A6 are associated with lipid metabolism and cardiovascular risk in a Chinese population. J. Lipid Res. 54 (11), 3122–3130. 10.1194/jlr.M041186

[B63] Li Z. Liao X. Hu Y. Li M. Tang M. Zhang S. (2023a). SLC27A4-mediated selective uptake of mono-unsaturated fatty acids promotes ferroptosis defense in hepatocellular carcinoma. Free Radical Biol. Medicine 201, 41–54. 10.1016/j.freeradbiomed.2023.03.013 36924851

[B64] Li X. Wang J. Guo Z. Ma Y. Xu D. Fan D. (2023b). Copper metabolism-related risk score identifies hepatocellular carcinoma subtypes and SLC27A5 as a potential regulator of cuproptosis. Aging 15 (24), 15084–15113. 10.18632/aging.205334 38157255 PMC10781498

[B65] Li X. Liu J. Jing Z. Li S. (2025). SLC27A3 downregulation restores Th17/Treg balance and alleviates COPD *via* JAK2/STAT3 pathway inhibition. Allergologia Immunopathol. 53 (1), 91–98. 10.15586/aei.v53i1.1215 39786880

[B66] Lin M. H. Chang K. W. Lin S. C. Miner J. H. (2010). Epidermal hyperproliferation in mice lacking fatty acid transport protein 4 (FATP4) involves ectopic EGF receptor and STAT3 signaling. Dev. Biol. 344 (2), 707–719. 10.1016/j.ydbio.2010.05.020 20513444 PMC2914132

[B67] Liu K. T. Yeh I. J. Chou S. K. Yen M. C. Kuo P. L. (2018). Regulatory mechanism of fatty acid-CoA metabolic enzymes under endoplasmic reticulum stress in lung cancer. Oncol. Rep. 40 (5), 2674–2682. 10.3892/or.2018.6664 30132556

[B68] Liu C. Wang J. Li D. Ni R. Zhao M. Huang C. (2022). Solute carrier family 27 member 6 (SLC27A6) possibly promotes the proliferation of papillary thyroid cancer by regulating c-MYC. Biochem. Genetics 60 (6), 2313–2326. 10.1007/s10528-022-10218-3 35348939

[B69] Liu Z. Yuan J. Zeng Q. Wu Z. Han J. (2024). UBAP2 contributes to radioresistance by enhancing homologous recombination through SLC27A5 ubiquitination in hepatocellular carcinoma. Biochimica Biophysica Acta. Mol. Basis Dis. 1870 (8), 167481. 10.1016/j.bbadis.2024.167481 39186963

[B70] Lobo S. Wiczer B. M. Bernlohr D. A. (2007). Functional analysis of long-chain acyl-CoA synthetase 1 in 3T3-L1 adipocytes. J. Biol. Chem. 282 (8), 5458–5467. 10.1074/jbc.M609342200 PMC2709349 19429676

[B71] Lu D. Li Y. Niu X. Sun J. Zhan W. Shi Y. (2024a). STAT2/SLC27A3/PINK1-mediated mitophagy remodeling lipid metabolism contributes to pazopanib resistance in clear cell renal cell carcinoma. Research (Wash D C) 7, 0539. 10.34133/research.0539 39600540 PMC11588985

[B72] Lu L. Li J. Zheng Y. Luo L. Huang Y. Hu J. (2024b). High expression of SLC27A2 predicts unfavorable prognosis and promotes inhibitory immune infiltration in acute lymphoblastic leukemia. Transl. Oncol. 45, **101952**. 10.1016/j.tranon.2024.101952 PMC11053221 38640787

[B73] Maekawa M. Iwayama Y. Ohnishi T. Toyoshima M. Shimamoto C. Hisano Y. (2015). Investigation of the Fatty acid transporter-encoding genes SLC27A3 and SLC27A4 in autism. Sci. Rep. 5, 16239. 10.1038/srep16239 26548558 PMC4637822

[B74] Magi A. Masselli M. Sala C. Guerriero A. Laise P. Puccini B. (2019). The ion channels and transporters gene expression profile indicates a shift in excitability and metabolisms during malignant progression of Follicular Lymphoma. Sci. Rep. 9 (1), 8586. 10.1038/s41598-019-44661-x 31197180 PMC6565741

[B75] Mandaviya P. R. Aïssi D. Dekkers K. F. Joehanes R. Kasela S. Truong V. (2017). Homocysteine levels associate with subtle changes in leukocyte DNA methylation: an epigenome-wide analysis. Epigenomics 9 (11), 1403–1422. 10.2217/epi-2017-0038 28990796

[B76] Mao X. Liu T. Yu S. Wei Y. Zhou C. Kuai X. (2025). CEACAM6 facilitates gastric cancer progression through upregulating SLC27A2. Cancer Gene Ther. 32 (1), 51–60. 10.1038/s41417-024-00846-9 39562695

[B77] Melton E. M. Cerny R. L. Watkins P. A. DiRusso C. C. (2011). Human fatty acid transport protein 2a/very long chain acyl-CoA synthetase 1 (FATP2a/VLCS1) has distinct roles in fatty acid transport and activation. J. Biol. Chem. 286 (35), 30670–30679. 10.1074/jbc.M111.258137 21768100 PMC3162428

[B78] Mendes C. Lopes-Coelho F. Ramos C. Martins F. Santos I. Rodrigues A. (2019). Unraveling SLC27A1/FATP1, regulated by ER-β, as a targeted breast cancer innovative therapy. Sci. Rep. 9 (1), 14107. 10.1038/s41598-019-50531-3 31575907 PMC6773857

[B79] Meng C. Sun Y. Liu G. (2023). Establishment of a prognostic model for ovarian cancer based on mitochondrial metabolism-related genes. Front. Oncol. 13, 1144430. 10.3389/fonc.2023.1144430 37256178 PMC10226651

[B80] Miner G. E. Gylling K. R. Smith M. D. (2023). SLC27A1/FATP1 interacts with PLIN5 at membrane contact sites to facilitate lipid droplet-to-mitochondria fatty acid transport in brown adipocytes. Nat. Metab. 5 (3), 487–499. 10.1038/s42255-023-00756-2

[B81] Mishima T. Miner J. H. Morizane M. Stahl A. Sadovsky Y. (2011). The expression and function of fatty acid transport protein-6 in human trophoblast cells. Placenta 32 (10), 801–807. 10.1016/j.placenta.2011.07.082 PMC3197585 22028793

[B82] Mitchell R. W. On N. H. Del Bigio M. R. Miller D. W. Hatch G. M. (2011). Fatty acid transport protein expression in human brain and potential role in fatty acid transport across human brain microvessel endothelial cells. J. Neurochem. 117 (4), 735–746. 10.1111/j.1471-4159.2011.07245.x 21395585

[B83] Mitsuyoshi H. Yasui K. Harano Y. Endo M. Tsuji K. Minami M. (2009). Hepatic SLC27A5 expression is upregulated in patients with nonalcoholic fatty liver disease and correlates with metabolic syndrome components. Hepatol. Res. 39 (11), 1078–1085. 10.1111/j.1872-034X.2009.00556.x

[B84] Mohammed A. Janakiram N. B. Suen C. Stratton N. Lightfoot S. Singh A. (2019). Targeting cholecystokinin-2 receptor for pancreatic cancer chemoprevention. Mol. Carcinogenesis 58 (10), 1908–1918. 10.1002/mc.23084 31313401 PMC6721979

[B85] Moulson C. L. Lin M. H. White J. M. Newberry E. P. Davidson N. O. Miner J. H. (2007). Keratinocyte-specific expression of fatty acid transport protein 4 rescues the wrinkle-free phenotype in Slc27a4/Fatp4 mutant mice. J. Biol. Chem. 282 (21), 15912–15920. 10.1074/jbc.M701779200 17401141

[B86] Nath A. Li I. Roberts L. R. Chan C. (2021). SLC27A1-mediated lipid accumulation drives invasiveness and chemoresistance in breast and pancreatic cancers. Oncogene 40 (12), 2145–2157. 10.1038/s41388-021-01702-3

[B88] Nickerson J. G. Alkhateeb H. Benton C. R. Lally J. Nickerson J. Han X. X. (2009). Greater transport efficiencies of the membrane fatty acid transporters FAT/CD36 and FATP4 compared with FABPpm and SLC27A1/FATP1 and differential effects on fatty acid esterification and oxidation in rat skeletal muscle. J. Biol. Chem. 284 (24), 16522–16530. 10.1074/jbc.M109.004788 19380575 PMC2713524

[B89] Niculae A. M. Dobre M. Herlea V. Vasilescu F. Ceafalan L. C. Trandafir B. (2022). Lipid handling protein gene expression in colorectal cancer: CD36 and targeting miRNAs. Int. J. Mol. Sci. 23 (24), 15618. 10.3390/ijms232415618 36556492 PMC9786157

[B90] Ochiai Y. Uchida Y. Ohtsuki S. Tachikawa M. Aizawa S. Terasaki T. (2017). The blood-brain barrier fatty acid transport protein 1 (SLC27A1/FATP1/SLC27A1) supplies docosahexaenoic acid to the brain, and insulin facilitates transport. J. Neurochem. 141 (3), 400–412. 10.1111/jnc.13943 28035674

[B91] Ochiai Y. Uchida Y. Tachikawa M. Couraud P. O. Terasaki T. (2019). Amyloid beta25-35 impairs docosahexaenoic acid efflux by down-regulating fatty acid transport protein 1 (SLC27A1/FATP1/SLC27A1) protein expression in human brain capillary endothelial cells. J. Neurochem. 150 (4), 385–401. 10.1111/jnc.14722 31091338

[B92] Park D. Kim E. Lee H. Shin E. A. Lee H. Lee J. W. (2021). Tetraspanin TM4SF5 in hepatocytes negatively modulates SLC27A transporters during acute fatty acid supply. Arch. Biochem. Biophys. 710, 109004. 10.1016/j.abb.2021.109004 34364885

[B93] Poenaru R. C. Milanesi E. Niculae A. M. Dobre A. M. Vladut C. Ciocîrlan M. (2025). Dysregulation of genes involved in the long-chain fatty acid transport in pancreatic ductal adenocarcinoma. World J. Gastroint. Oncol. 17 (1), 98409. 10.4251/wjgo.v17.i1.98409 PMC11664611 39817147

[B94] Poreba M. A. Dong C. X. Li S. K. Stahl A. Miner J. H. Brubaker P. L. (2012). Role of fatty acid transport protein 4 in oleic acid-induced glucagon-like peptide-1 secretion from murine intestinal L cells. Am. J. Physiol. Endocrinol. Metabolism 303 (7), E899–E907. 10.1152/ajpendo.00116.2012 22871340 PMC3469616

[B95] Sebastián D. Guitart M. García-Martínez C. Mauvezin C. Orellana-Gavaldà J. M. Serra D. (2009). Novel role of SLC27A1/FATP1 in mitochondrial fatty acid oxidation in skeletal muscle cells. J. Lipid Res. 50 (9), 1789–1799. 10.1194/jlr.M800535-JLR200 19429947 PMC2724792

[B96] Schaffer J. E. Lodish H. F. (1994). Expression cloning and characterization of a novel adipocyte long chain fatty acid transport protein. Cell 79 (3), 427–436. 10.1016/0092-8674(94)90252-6 7954810

[B97] Schmuth M. Ortegon A. M. Stahl A. Moosbrugger-Martinz V. (2005a). Differential expression of fatty acid transport proteins in epidermis and skin appendages. J. Investi. Dermatol. 125 (6), 1170–1175. 10.1111/j.0022-202X.2005.23934.x 16354187

[B98] Schmuth M. Ortegon A. M. Jiang Y. J. Elias P. M. Stahl A. (2005b). Thematic review series: skin lipids. The role of epidermal lipids in cutaneous permeability barrier homeostasis. J. Lipid Res. 46 (12), 2517–2524. 10.1194/jlr.R500013-JLR200 17872588

[B99] Seessle J. Liebisch G. Staffer S. Tuma-Kellner S. Merle U. Herrmann T. (2024). Enterocyte-specific FATP4 deficiency elevates blood lipids *via* a shift from polar to neutral lipids in distal intestine. Am. J. Physiol. Gastrointest. Liver Physiol. 327 (2), G202–G216. 10.1152/ajpgi.00109.2024 38915276

[B100] Serrano A. Asnani-Kishnani M. Couturier C. Astier J. Palou A. Landrier J. F. (2020). DNA methylation changes are associated with the programming of white adipose tissue browning features by resveratrol and nicotinamide riboside neonatal supplementations in mice. Nutrients 12 (2), 461. 10.3390/nu12020461 32059412 PMC7071331

[B101] Shang K. Ma N. Che J. Li H. Hu J. Sun H. (2023). SLC27A2 mediates FAO in colorectal cancer through nongenic crosstalk regulation of the PPARs pathway. BMC Cancer 23 (1), 335. 10.1186/s12885-023-10816-3 37041476 PMC10091540

[B102] Shim J. Moulson C. L. Newberry E. P. Lin M. H. Xie Y. Humphreys J. M. (2009). Fatty acid transport protein 4 is dispensable for intestinal lipid absorption in mice. J. Lipid Res. 50 (3), 491–500. 10.1194/jlr.M800400-JLR200 18843142 PMC2638106

[B103] Shubbar E. Kovács A. Hajizadeh S. Parris T. Z. Nemes S. Gunnarsdóttir K. (2013). Elevated cyclin B2 expression in invasive breast carcinoma is associated with unfavorable clinical outcome. BMC Cancer 13, 1. 10.1186/1471-2407-13-1 23282137 PMC3545739

[B104] Silveira S. M. Villacis R. A. Marchi F. A. Barros Filho M. deC. Drigo S. A. Neto C. S. (2013). Genomic signatures predict poor outcome in undifferentiated pleomorphic sarcomas and leiomyosarcomas. PloS One 8 (6), e67643. 10.1371/journal.pone.0067643 23825676 PMC3692486

[B105] Stahl A. (2004). A current review of fatty acid transport proteins (SLC27). Pflugers Archiv Eur. J. Physiol. 447 (5), 722–727. 10.1007/s00424-003-1106-z 12856180

[B106] Stahl A. Evans J. G. Pattel S. Hirsch D. Lodish H. F. (2002). Insulin causes fatty acid transport protein translocation and enhanced fatty acid uptake in adipocytes. Dev. Cell 2 (4), 477–488. 10.1016/S1534-5807(02)00143-0 11970897

[B107] Su J. Wu S. Tang W. Qian H. Zhou H. Guo T. (2016). Reduced SLC27A2 induces Cisplatin resistance in lung cancer stem cells by negatively regulating Bmi1-ABCG2 signaling. Mol. Carcinog. 55 (11), 1822–1832. 10.1002/mc.22430 26513225

[B108] Supti S. T. Koehn L. M. Newman S. A. Pan Y. Nicolazzo J. A. (2024). Iron reduces the trafficking of Fatty acids from human immortalised brain microvascular endothelial cells through modulation of Fatty acid transport protein 1 (SLC27A1/FATP1/SLC27A1). Pharm. Res. 41 (8), 1631–1648. 10.1007/s11095-024-03743-w 39044044 PMC11362236

[B109] Tao L. Mohammad M. A. Milazzo G. Moreno-Smith M. Patel T. D. Zorman B. (2022). MYCN-driven fatty acid uptake is a metabolic vulnerability in neuroblastoma. Nat. Commun. 13 (1), 3728. 10.1038/s41467-022-31331-2 35764645 PMC9240069

[B110] Tao J. Liu Y. Tang X. Nie D. Wu K. Wang K. (2025). Hypoxia reduces SLC27A5 to promote hepatocellular carcinoma proliferation by repressing HNF4A. Biochim. Biophys. Acta. Mol. Cell Res. 1872 (3), 119916. 10.1016/j.bbamcr.2025.119916 39938688

[B111] Tharp K. M. Khalifeh-Soltani A. Park H. M. Yurek D. A. Falcon A. Wong L. (2016). Prevention of gallbladder hypomotility *via* FATP2 inhibition protects from lithogenic diet-induced cholelithiasis. Am. J. Physiol. Gastrointest. Liver Physiol 310 (10), G855–G864. 10.1152/ajpgi.00316.2015 27033116 PMC4888547

[B112] Torelli Hijo A. H. Coutinho C. P. Alba-Loureiro T. C. Moreira Leite J. S. Bargi-Souza P. Goulart-Silva F. (2019). High fat diet modulates the protein content of nutrient transporters in the small intestine of mice: possible involvement of PKA and PKC activity. Heliyon 5 (10), e02611. 10.1016/j.heliyon.2019.e02611 31667423 PMC6812199

[B113] Uchiyama A. Aoyama T. Kamijo K. Uchida Y. Kondo N. Orii T. (1996). Molecular cloning of cDNA encoding rat very long-chain acyl-CoA synthetase. J. Biol. Chem. 271 (48), 30360–30365. 10.1074/jbc.271.48.30360 8939997

[B114] Verma S. Shankar E. Chan E. R. Gupta S. (2020). Metabolic reprogramming and predominance of solute carrier genes during acquired enzalutamide resistance in prostate cancer. Cells 9 (12), 2535. 10.3390/cells9122535 33255236 PMC7759897

[B115] Viraragavan A. Willmer T. Patel O. Basson A. Johnson R. Pheiffer C. (2021). Cafeteria diet induces global and Slc27a3-Specific hypomethylation in Male Wistar rats. Adipocyte 10 (1), 108–118. 10.1080/21623945.2021.1886697 33570456 PMC7889207

[B116] Wang H. Lee J. H. Tian Y. (2019a). Critical Genes in White adipose tissue based on gene expression profile following exercise. Int. J. Sports Med. 40 (1), 57–61. 10.1055/a-0768-7866 30497092

[B117] Wang Y. Cheng T. Lu M. Mu Y. Li B. Li X. (2019b). TMT-based quantitative proteomics revealed follicle-stimulating hormone (FSH)-related molecular characterizations for potentially prognostic assessment and personalized treatment of FSH-positive non-functional pituitary adenomas. EPMA J. 10 (4), 395–414. 10.1007/s13167-019-00187-w 31832114 PMC6882982

[B118] Wang Y. Chen Y. Zhu B. Ma L. Xing Q. (2021). A novel nine apoptosis-related genes signature predicting overall survival for Kidney Renal clear cell carcinoma and its associations with immune infiltration. Front. Mol. Biosci. 8, 567730. 10.3389/fmolb.2021.567730 33748185 PMC7969794

[B119] Wang J. Qiao Y. Sun H. Chang H. Zhao H. Zhang S. (2022). Decreased SLC27A5 suppresses lipid synthesis and tyrosine metabolism to activate the cell cycle in hepatocellular carcinoma. Biomedicines 10 (2), 234. 10.3390/biomedicines10020234 35203444 PMC8869743

[B120] Wang J. Zhao S. Sun J. Wang X. Guan M. Yin J. (2023). Oncogenic role and potential regulatory mechanism of fatty acid binding protein 5 based on a pan-cancer analysis. Sci. Rep. 13 (1), 4060. 10.1038/s41598-023-30695-9 36906605 PMC10008585

[B121] Wang Y. Chen X. Li Y. Zhang Z. Xia L. Jiang J. (2024). SLC27A2 is a potential immune biomarker for hematological tumors and significantly regulates the cell cycle progression of diffuse large B-Cell lymphoma. BMC Med. Genomics 17 (1), 105. 10.1186/s12920-024-01853-3 38664735 PMC11046844

[B122] Wu Q. Ortegon A. M. Tsang B. Doege H. Feingold K. R. Stahl A. (2006a). SLC27A1/FATP1 is an insulin-sensitive fatty acid transporter involved in diet-induced obesity. Mol. Cellular Biol. 26 (9), 3455–3467. 10.1128/MCB.26.9.3455-3467.2006 16611988 PMC1447434

[B123] Wu Q. Kazantzis M. Doege H. Ortegon A. M. Tsang B. Falcon A. (2006b). Fatty acid transport protein 1 is required for nonshivering thermogenesis in brown adipose tissue. Diabetes 55 (12), 3229–3237. 10.2337/db06-0749 17130465

[B124] Wu Y. Pi D. Chen Y. Zuo Q. Zhou S. Ouyang M. (2022). Ginsenoside Rh4 inhibits colorectal cancer cell proliferation by inducing ferroptosis *via* autophagy activation. Evid. Based Complement. Altern. Med. 2022, 6177553-19. 10.1155/2022/6177553 PMC9168088 35677385

[B125] Wu Y. Pi D. Zhou S. Yi Z. Dong Y. Wang W. (2023). Ginsenoside Rh3 induces pyroptosis and ferroptosis through the Stat3/p53/NRF2 axis in colorectal cancer cells. Acta Biochim. Biophys. Sin. (Shanghai) 55 (4), 587–600. 10.3724/abbs.2023068 37092860 PMC10195137

[B126] Xu W. Chen Z. Liu G. Dai Y. Xu X. Ma D. (2021). Identification of a potential PPAR-Related multigene signature predicting prognosis of patients with hepatocellular carcinoma. PPAR Res. 2021, 6642939-10. 10.1155/2021/6642939 33777129 PMC7981186

[B127] Xu N. Xiao W. Meng X. Li W. Wang X. Zhang X. (2022). Up-regulation of SLC27A2 suppresses the proliferation and invasion of renal cancer by down-regulating CDK3-mediated EMT. Cell Death Discov. 8 (1), 351. 10.1038/s41420-022-01145-8 35927229 PMC9352701

[B128] Xu F. L. Wu X. H. Chen C. Wang K. Huang L. Y. Xia J. (2023). SLC27A5 promotes sorafenib-induced ferroptosis in hepatocellular carcinoma by downregulating glutathione reductase. Cell Death Dis. 14 (1), 22. 10.1038/s41419-023-05558-w 36635256 PMC9837139

[B129] Xu X. Li A. X. Wang C. C. Zhang H. J. Lou Y. F. Davies A. (2025). Expression of SLC27A5, a long chain fatty acid transporter, in clinical breast cancer and the clinical value in assessing the outcome and metastasis. Mol. Oncol. 19 (S1), 929.

[B130] Yang Y. Yang X. Ren S. Cao Y. Wang Z. Cheng Z. (2024). Identification and analysis of prognostic metabolic characteristics in colon adenocarcinoma. Heliyon 10 (6), e27388. 10.1016/j.heliyon.2024.e27388 38509965 PMC10950572

[B131] Yen M. C. Chou S. K. Kan J. Y. Kuo P. L. Hou M. F. Hsu Y. L. (2018). Solute carrier family 27 member 4 (SLC27A4) enhances cell growth, migration, and invasion in breast cancer cells. Int. J. Mol. Sci. 19 (11), 3434. 10.3390/ijms19113434 30388870 PMC6274775

[B132] Yen M. C. Chou S. K. Kan J. Y. Kuo P. L. Hou M. F. Hsu Y. L. (2019). New Insight on solute carrier family 27 member 6 (SLC27A6) in tumoral and non-tumoral breast cells. Int. J. Med. Sci. 16 (3), 366–375. 10.7150/ijms.29946 30911270 PMC6428986

[B133] Yeung C. L. S. Ng T. H. Lai C. J. Xue T. Mao X. Tey S. K. (2025). Small extracellular vesicle-derived nicotinamide phosphoribosyltransferase (NAMPT) induces acyl-coenzyme A synthetase SLC27A4-Mediated glycolysis to promote hepatocellular carcinoma. J. Extracell. Vesicles 14 (4), e70071. 10.1002/jev2.70071 40237223 PMC12000932

[B134] Zaidi N. Lupien L. Kuemmerle N. B. Kinlaw W. B. Swinnen J. V. Smans K. (2013). Lipogenesis and lipolysis: the pathways exploited by the cancer cells to acquire fatty acids. Prog. Lipid Res. 52 (4), 585–589. 10.1016/j.plipres.2013.08.005 24001676 PMC4002264

[B135] Zhang M. Di Martino J. S. Bowman R. L. Campbell N. R. Baksh S. C. Simon-Vermot T. (2018). Adipocyte-Derived lipids mediate melanoma progression *via* FATP proteins. Cancer Dis. 8 (8), 1006–1025. 10.1158/2159-8290.CD-17-1371 29903879 PMC6192670

[B136] Zhang H. Shen Z. Yang Z. Jiang H. Chu S. Mao Y. (2021a). Abundance of solute carrier family 27 member 6 (SLC27A6) in the bovine mammary gland alters fatty acid metabolism. Food Funct. 12 (11), 4909–4920. 10.1039/d0fo03289a 34100479

[B137] Zhang F. Xue M. Jiang X. Yu H. Qiu Y. Yu J. (2021b). Identifying SLC27A5 as a potential prognostic marker of hepatocellular carcinoma by weighted gene co-expression network analysis and *in vitro* assays. Cancer Cell Internat. 21 (1), 174. 10.1186/s12935-021-01871-6 PMC7968262 33731144

[B138] Zhang X. Zhang Y. Wang Y. Li X. Chen G. (2022). Identification of SLC27A3 and STAU1 as novel diagnostic biomarkers for chronic obstructive pulmonary disease *via* machine learning analysis. Respir. Res. 23 (1), 147. 10.1186/s12931-022-02056-8 35672770

[B139] Zhang Y. Li J. Wang Q. Zhang X. Liu H. Zhu Y. (2023). Hypoxia-induced upregulation of SLC27A1 and SLC27A2 enhances fatty acid oxidation in tumor cells *via* HIF-1α signaling. Cancer Res. 83 (7), 1123–1135. 10.1158/0008-5472.CAN-22-1945

[B140] Zhang W. Liu J. Ren X. Zhang Z. Zhou M. Li Y. (2024a). Identification of the novel markers of PPAR signalling affecting immune microenvironment and immunotherapy response of lung adenocarcinoma patients. J. Cellular Mol. Med. 28 (5), e17877. 10.1111/jcmm.17877 37556076 PMC10902583

[B141] Zhang B. Zhang Y. Chang K. Hou N. Fan P. Ji C. (2024b). Risk assessment model based on nucleotide metabolism-related genes highlights SLC27A2 as a potential therapeutic target in breast cancer. J. Cancer Res. Clin. Oncol. 150 (5), 258. 10.1007/s00432-024-05754-x 38753091 PMC11098904

[B142] Zheng P. Mao Z. Luo M. Zhou L. Wang L. Liu H. (2023). Comprehensive bioinformatics analysis of the solute carrier family and preliminary exploration of SLC25A29 in lung adenocarcinoma. Cancer Cell Internat. 23 (1), 222. 10.1186/s12935-023-03082-7 PMC10543265 37775731

[B143] Zhu Y. Zhang J. Wang C. Zheng T. Di S. Wang Y. (2023). Ameliorative effect of ethanolic Echinacea purpurea against hyperthyroidism-induced oxidative stress *via* AMRK and PPAR signal pathway using transcriptomics and network pharmacology analysis. Int. J. Mol. Sci. 24 (1), 187. 10.3390/ijms24010187 PMC9820381 36613632

[B144] Zhu W. Zheng Y. Liu J. Zhao C. Sun N. Qu X. (2023). Analysis of Fatty acid metabolism in fetal and failing hearts by single-cell RNA sequencing revealed SLC27A6 as a critical gene in heart maturation. Acta Cardiol. Sin. 39 (4), 580–598. 10.6515/ACS.202307_39(4).20221219B 37456940 PMC10346055

